# An insight into misidentification of the small-subunit ribosomal RNA (18S rRNA) gene sequences of *Theileria* spp. as *Theileria annulata*

**DOI:** 10.1186/s12917-022-03540-w

**Published:** 2022-12-28

**Authors:** Anil Kumar Nehra, Ansu Kumari, Aman Dev Moudgil, Sukhdeep Vohra

**Affiliations:** 1 Department of Veterinary Parasitology, Lala Lajpat Rai University of Veterinary and Animal Sciences, 125004 Hisar, Haryana India; 2 Department of Veterinary Medicine, Lala Lajpat Rai University of Veterinary and Animal Sciences, 125004 Hisar, Haryana India

**Keywords:** *Theileria annulata*, 18S rRNA, Misidentification, *Theileria* spp., Genetic diversity, Sequence analysis

## Abstract

**Background:**

There had been isolated reports of the presence of novel *Theileria annulata* genotypes based on the 18S rRNA gene sequence data from India, Pakistan and Saudi Arabia; but, these studies were restricted to limited field samples. Additionally, no comparative study has been conducted on all the isolates of this parasite from different countries whose sequences are available in the nucleotide databases. Therefore, we aimed to study the genetic diversity of *T. annulata* based on all available nearly complete 18S rRNA gene sequences in the GenBank™. Out of a total of 312 gene sequences of *T. annulata* available in the NCBI database, only 70 nearly complete sequences (> 1527 bp) were used for multiple sequence alignment.

**Results:**

The maximum likelihood tree obtained using TN93 + G + I model manifested two major clades. All the valid host-cell transforming *Theileria* species clustered in one clade. The *T. annulata* designated sequences occupying this clade clustered together, excluding two isolates (DQ287944 and EU083799), and represented the true *T. annulata* sequences (*n* = 54). DQ287944 and EU083799 exhibited close association with *Theileria lestoquardi*. In addition, 14 Indian sequences formed a large monophyletic group with published *Theileria orientalis* sequences. The broad range of sequence identity (95.8–100%) of *T. annulata* designated sequences indicated the presence of different *Theileria* spp. A closer analysis revealed the presence of three *Theileria* spp., namely, *T. annulata*, *T. orientalis,* and two isolates (DQ287944 and EU083799) closely related to *T. lestoquardi*. The true *T. annulata* sequences manifested 98.8–100% nucleotide identity within them. EU083799 and 14 misidentified Indian *T. annulata* sequences exhibited the highest similarity with *T. lestoquardi* (98.6–98.8%) and *T. orientalis* (98.0-99.9%) in comparison with the other *Theileria* spp. of domestic and wild ruminants.

**Conclusion:**

In the course of analyzing the genetic diversity of *T. annulata*, we identified the nearly complete 18S rRNA gene sequences of other *Theileria* spp. that have not only been misidentified as *T. annulata* in the GenBank™, but are also published as *T. annulata*. Moreover, a high level of sequence conservation was noticed in the 18S rRNA gene of true *T. annulata* and *T. orientalis* sequences.

**Supplementary Information:**

The online version contains supplementary material available at 10.1186/s12917-022-03540-w.

## Background

Theileriosis is a tick-borne haemoprotozoan disease infecting a wide range of domestic and wild mammals. It has ubiquitous distribution due to the global existence of tick vectors [1]. There are many *Theileria* spp. that infect bovines; the most pathogenic and economically important are *T. parva*, which causes East Coast fever (ECF), *T. annulata*, which causes Tropical/ Mediterranean theileriosis, and *T. orientalis* (*T. orientalis*/*buffeli* group), which causes Oriental theileriosis or *Theileria*-associated bovine anaemia (TABA). Similarly, *Theileria lestoquardi*, which causes Malignant ovine theileriosis, *Theileria uilenbergi* and *Theileria luwenshuni* are the most pathogenic species of economic importance infecting small ruminants [1, 2]. Amongst the various diseases caused by the genus *Theileria*, bovine tropical theileriosis is an economically important disease of cattle and water buffaloes causing significant economic losses with several complications on both local and global scales [3–5]. It is caused by *Theileria annulata*, an intracellular apicomplexan parasite. The infected animals may present variable clinical symptoms, namely, fever, anemia, respiratory distress, jaundice, enlarged superficial lymph nodes, decreased milk yield, progressive loss of body weight, etc. [2]. The mild and subclinical infections are overlooked and animals remain carriers, but during stress conditions, the disease may flare up to a clinical state [6, 7]. The variable clinical presentation as well as irregular clinical signs and symptoms of the disease frequently noticed by the field veterinarians could be related to genetic changes in this protistan parasite [8], but this needs to be further investigated.

The different isolates of this apicomplexan parasite exhibit variable virulence in the susceptible bovine population in enzootic areas [9]. Additionally, the animals vaccinated with macroschizont attenuated cell culture vaccine developed from local isolates showed vaccination failures when challenged with a heterologous strain [10, 11], and it has been shown that such vaccines confer protection only against homologous isolates under field conditions [12]. Besides, the reduced efficacy of theilericidal drugs indicates emergence of drug resistant strains [13]. Taken together, all these attributes signify the presence of extensive genetic diversity in the said organism.

The commonly used genetic markers for *T*. *annulata* identification and characterization are the small-subunit ribosomal RNA (18S rRNA) gene [7, 14, 15], the *T*. *annulata* merozoites surface antigen *(Tams1*) encoding gene [16, 17], the β-tubulin gene [18], cytochrome b gene [19, 20] and the heat shock protein 70 encoding gene (HSP70) [21]. The 18S rRNA gene sequences have proven to be useful for deducing evolutionary patterns and are a widely used marker for genetic characterization, taxonomic classification and phylogenetic studies. It is used for determining species level infection of a number of parasites including *T. annulata* [22] and a number of sequences of different isolates from several parts of the world are freely available in the nucleotide databases for comparisons. The conserved and hypervariable (V4) regions of this gene are effectively studied and useful for elucidating relationships amongst different isolates and species [23]. The inter- and intra-species diversities can be estimated by the sequence and phylogenetic analyses based on this gene. There had been isolated reports of the presence of novel *T. annulata* genotypes based on the 18S rRNA gene sequence data from several countries, viz., India [24], Pakistan [25] and Saudi Arabia [26]; but, these studies were restricted to limited field samples. Additionally, no comparative study has been conducted on all the isolates of this parasite from different countries whose sequences are available in the nucleotide databases. Therefore, we aimed to study the genetic diversity of *T. annulata* based on all available nearly complete 18S rRNA gene sequences in the GenBank™. Further, an attempt was made to establish the phylogenetic relationship among all the sequences and with other species of the genus *Theileria* infecting domestic and wild ruminants.

## Results

### Phylogenetic analysis

**Fig. 1:**
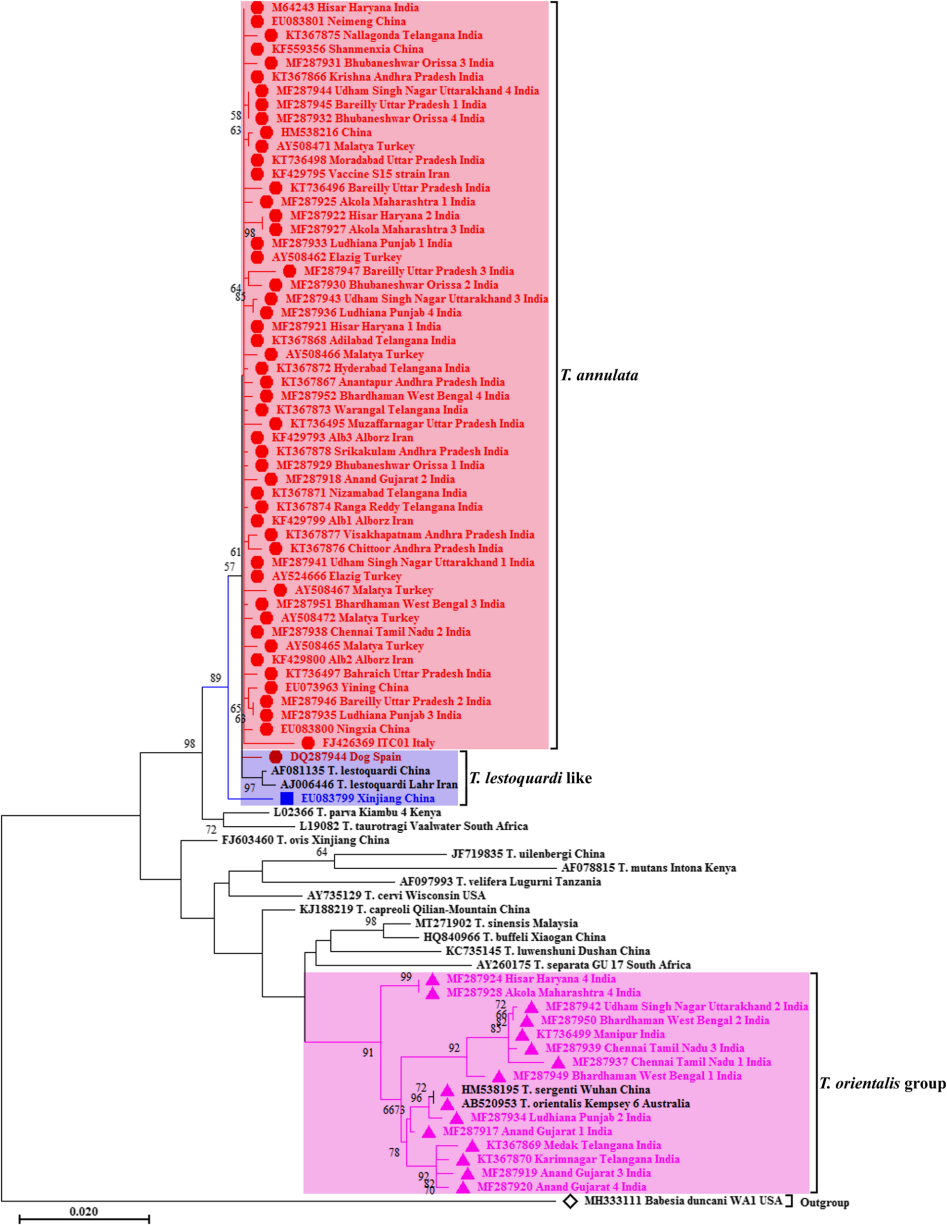
The phylogenetic tree of different isolates of *T. annulata* with other *Theileria* species infecting domestic and wild ruminants based on nearly complete nucleotide sequences of  the nuclear 18S rRNA gene. Tamura-Nei model (TN93 + G + I) of maximum likelihood method was applied for this analysis. The taxon name of each sequence employed is depicted by its accession number followed by the place of sampling, if any, and the country of origin. The detail of accession numbers used is provided in supplementary Table 1. The color coding is done as mentioned below. Red font color with red filled circles as taxon markers- sequences that are correctly identified as *T. annulata* in the GenBank™ (shaded light red); blue font color with blue filled square as taxon marker- sequence deposited in the GenBank™ as *T. annulata* but is more closely related to *T. lestoquardi* (shaded light blue); pink font color with pink filled triangles as taxon markers- sequences deposited in the GenBank™ as *T. annulata* but are more closely related to *T. orientalis* (shaded light pink); Default font color without any taxon marker- rest of the sequences including outgroup

Out of a total of 312 small-subunit ribosomal RNA gene sequences of *T. annulata* available in the database, only 70 nearly complete sequences (> 1527 bp) were used for multiple sequence alignment to construct a phylogenetic tree. The maximum likelihood tree obtained using TN93 + G + I model manifested two major clades (Fig. 1). All the valid host-cell transforming *Theileria* species (*T. parva*, *T. annulata*, *T. lestoquardi* and *T. taurotragi*) clustered in one clade. The *T. annulata* designated sequences occupying this clade revealed close association with *T. lestoquardi* (AF081135, China; AJ006446, Iran), *T. parva* (L02366, Kenya) and *T. taurotragi* (L19082, South Africa), which are all leucocyte transforming *Theileria* parasites. These *T. annulata* designated sequences clustered together, excluding two isolates (DQ287944 and EU083799), and represented the true *T. annulata* sequences. The Spanish (DQ287944, dog) and Chinese (EU083799) isolates exhibited close association with *T. lestoquardi* (AF081135, China; AJ006446, Iran).

In addition, 14 nearly complete 18S rRNA sequences deposited in the GenBank™ as *T. annulata* shared a major clade with all the non-transforming *Theileria* species. They formed a large monophyletic group with published *T. orientalis* sequences from Australia (AB520953) and China (HM538195). Thus, these Indian sequences represent *T. orientalis* which have been misidentified as *T. annulata* in the GenBank™ and are published as *T. annulata* in two separate studies by Kundave et al. [14] and George et al. [24] as described in Table 1.

**Table 1:** List of misidentified 18S rRNA gene sequences of *T. annulata* recognized in the present study

Accession No.	Parasite identification in the GenBank™	Parasite identification in research articles	Reference	Parasite identification in the current study
MF287942	*T. annulata*	*T. annulata*	[14]	*T. orientalis*
MF287917	*T. annulata*	*T. orientalis*	[14]	*T. orientalis*
MF287919	*T. annulata*	*T. annulata*	[14]	*T. orientalis*
MF287920	*T. annulata*	*T. annulata*	[14]	*T. orientalis*
MF287924	*T. annulata*	*T. orientalis*	[14]	*T. orientalis*
MF287934	*T. annulata*	*T. annulata*	[14]	*T. orientalis*
MF287928	*T. annulata*	*T. orientalis*	[14]	*T. orientalis*
MF287937	*T. annulata*	*T. annulata*	[14]	*T. orientalis*
MF287939	*T. annulata*	*T. annulata*	[14]	*T. orientalis*
MF287949	*T. annulata*	*T. annulata*	[14]	*T. orientalis*
MF287950	*T. annulata*	*T. annulata*	[14]	*T. orientalis*
KT736499	*T. annulata*	Unpublished	Shahzad M, Ram H, Kumar S, Chauhan RP, Sharma AK, Garg R, Goswami TK, Tiwari AK, Banerjee PS (Unpublished)	*T. orientalis*
KT367869	*T. annulata*	New genotypes of *T. annulata*	[24]	*T. orientalis*
KT367870	*T. annulata*	New genotypes of *T. annulata*	[24]	*T. orientalis*
EU083799	*T. annulata*	Unpublished	Liu A, Yin H, Luo J (Unpublished)	*T. lestoquardi*^a^

^a^Additional morphological, molecular and biological studies are required to confirm the species level identification

### Genetic diversity

The sequence identity in NCBI-BLAST showed that all the *T. annulata* designated sequences were 95.8–100% identical with each other, whereas 94.1–99.7% identical with the other *Theileria* species infecting ruminants. Sequence variations were detected in both conserved as well as in the hypervariable regions of the 18S rRNA gene at discrete places upon sequence alignment, indicating the presence of different parasite populations. A closer analysis revealed the presence of three distinct *Theileria* populations, namely, *T. annulata*, *T. orientalis* and two isolates (DQ287944 and EU083799) exhibiting close association with *T. lestoquardi*.

#### *Theileria annulata*

The 54 18S rRNA gene sequences of *T. annulata* originating from different countries, viz., India, China, Turkey and Iran, including the Iranian S15 vaccine strain (KF429795), manifested 98.8–100% nucleotide identity within them. In comparison with the other *Theileria* spp. infecting ruminants, they showed highest nucleotide identity (98.8–99.6%) with *T. lestoquardi* originating from China (AF081135) and Iran (AJ006446). The nucleotide variations within this group were recorded at 94 places throughout the alignment (Supplementary File 1).

#### *Theileria orientalis*

The 14 Indian small subunit ribosomal RNA sequences deposited in the GenBank™ as *T. annulata* but more closely related to *T. orientalis*, manifested 98–100% nucleotide identity amongst each other. On juxtaposition with the other *Theileria* spp. included in the study, they showed highest similarity (98.0–99.9%) with *T. orientalis*. The sequence variations were observed at 70 and 70–74 places within this group and when compared with the published *T. orientalis* sequences, respectively (Supplementary File 2).

#### *Theileria lestoquardi* like sequences

The only dog isolate of *T. annulata* (DQ287944, Spain) manifested 99.3% and 99.4% identity with *T. lestoquardi* of Iran (AJ006446) and China (AF081135), respectively; however, on additional amplification and sequencing of partial cytochrome b gene, it was confirmed to be *T. annulata* [27].

**Fig. 2:**
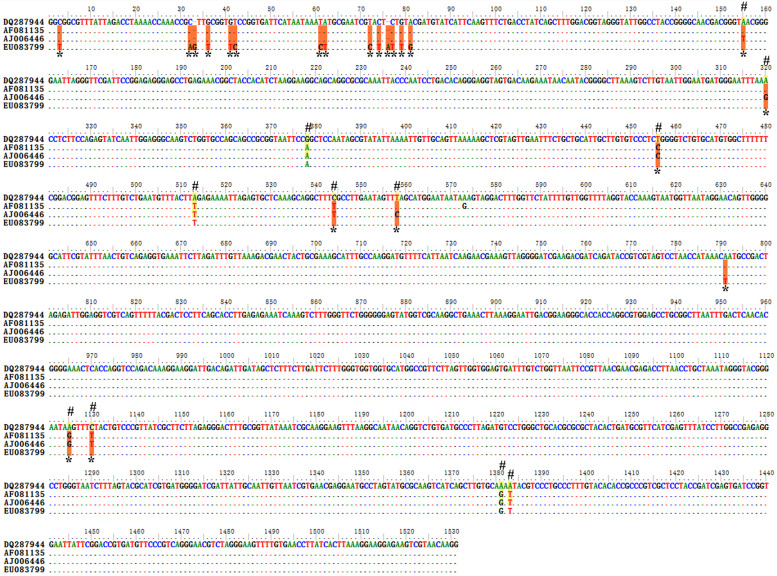
Sequence variations detected in the conserved and hypervariable regions of the 18S rRNA gene upon multiple sequence alignment of *T. lestoquardi* like sequences. DQ287944 (Spain) and EU083799 (China) exhibited sequence variations at seven (marked # and shaded yellow) and 22 places (marked * and shaded orange) when compared with the vaccine strain of *T. lestoquardi* (AJ006446; Iran), respectively

The 18S rRNA sequence of EU083799 (China) showed 98.6% and 98.8% identity with Iranian vaccine strain (AJ006446) and Chinese isolate (AF081135) of *T. lestoquardi*, respectively. It exhibited sequence variations at 22 places when compared with the vaccine strain of *T. lestoquardi* (AJ006446; Iran). The sequence and phylogenetic analyses suggest that it may be *T. lestoquardi* (Fig. 2).

The details of misidentified 18S rRNA gene sequences available in the GenBank™ as *T. annulata* and published as either *T. annulata* or *T. orientalis* are given in Table 1.

## Discussion

The genetic characterization of *T. annulata* based on the 18S rRNA gene has been described by several workers from different parts of the globe [7, 14, 24, 25, 28, 29]. The 18S rRNA sequencing approach has been widely used to distinguish between apicomplexan and other eukaryotic species due to a high copy number of 18S rRNA molecules in the ribosomes, besides existence of the hypervariable regions within highly conserved DNA sequences [23]. These regions have been thoroughly studied and are critical in determining evolutionary patterns and similarity amongst *Theileria* species [22]. Simultaneously, this gene has been extensively used for molecular and phylogenetic analyses of several *Theileria* species infecting livestock [30, 31].

For developing new vaccines, diagnostics, drugs, and designing effective theileriosis control strategies, it is essential to investigate the genetic diversity of the causative agent [7]. The broad range of percent identity (95.8–100%) between 18S rRNA sequences deposited as *T. annulata* in the GenBank™ was not in agreement with the expected sequence identity within a *Theileria* species and suggested the presence of different parasite populations. However, this wide range of sequence identity was in correlation with Kundave et al. [14] and George et al. [24], but contrary to the percent identity scores of Khan et al. [25] and Sivakumar et al. [32]. It was because both of the former studies included the misidentified sequences of *Theileria* spp. as *T. annulata*. The high level of sequence conservation was identified within the true 18S rRNA gene sequences of *T. annulata* (98.8–100%) and *T. orientalis* (98–100%). It was not only consistent with the expected 18S rRNA sequence identity within a *Theileria* species, but also supported the findings of previous studies [8, 15, 25, 29, 32, 33]. Only 18S rRNA sequences longer than 1527 bp were used for sequence and phylogenetic analyses because it is difficult to differentiate the truncated 18S rRNA gene sequences of *T. annulata* and *T. lestoquardi* on the basis of partial sequences, and the phylogenies constructed with the long 18S rRNA sequences are characterized by high bootstrap support for clades [32, 34]. The presence of transforming *Theileria* species, viz., *T. parva*, *T. annulata*, *T. lestoquardi* and *T. taurotragi*, in one clade reinforced the previous finding that these species evolved from a common ancestor [35, 36]. Although the present study was aimed to analyze the genetic diversity of *T. annulata*, the results of phylogenetic and sequence analyses indicated the misidentification of *Theileria* spp. sequences as *T. annulata* in the GenBank™.

Molecular methods, viz., polymerase chain reaction (PCR) and sequencing, are very useful tools for species level identification of *Theileria* spp. [7, 28, 29, 37, 38]. However, an incorrect taxon identification can be made if the custom DNA sequences are not carefully analyzed and compared with the data available in public sequence databases. It becomes particularly important when the DNA shows high sequence identity between two or more parasites.

The 18S rRNA sequences of different *Theileria* species share high sequence identities between them due to recent speciation events [39]. *Theileria lestoquardi* is perhaps recently evolved from *T. annulata* and their 18S rRNA sequences share high percent identity between them. The V4 hypervariable region of the 18S rRNA gene of both the parasites differs from each other by only three nucleotides. Similarly, the 18S rRNA sequences of *Theileria parva* and *Theileria* sp. (buffalo) are highly conserved and differ by only 11 nucleotides [33]. This high level of sequence similarity can result in misannotation of the original sequences, as has been observed in the present study. To avoid misannotation of sequences, it is important to conduct an inter-species comparison among different *Theileria* species followed by an intra-species analysis based on the percent identity and sequence length, before depositing any 18S rRNA sequence of *Theileria* species in a nucleotide database [40].

While the Chinese 18S rRNA sequence EU083799 group most closely with other *T. lestoquardi* sequences, this does not confirm that this parasite is *T. lestoquardi*. Further, morphological, molecular and biological studies are required to confirm that this parasite is indeed *T. lestoquardi*.

As most of the existing detection methods based on the amplification of the 18S rRNA gene, viz., conventional and real-time PCR assays, reverse line blot hybridization and LAMP, for different *Theileria* species are based on the assumption that the region of this gene targeted by the primers and/or probes is conserved among all the members of a species [39]. So, with the presence of sequence variations in both conserved and hypervariable regions of the 18S rRNA gene within and between *Theileria* species, it needs to be ascertained whether these tests would correctly identify the different sequences and distinguish between the different species.

Although the 18S rRNA gene is undoubtedly a useful marker for defining *Theileria* species, it has been demonstrated that the 18S rRNA sequences alone are not definitive for species level identification of some *Theileria* species, viz., *T. annulata* and *T. lestoquardi* [32, 39], *T. annulata* and *Theileria* sp. Yokoyama [32], and *T. parva* and *T.* sp. (buffalo) [41]. Mans and co-workers [33] have provided evidence for the existence of intermediate 18S rRNA gene sequences between *T. parva* and *T.* sp. (buffalo). Therefore, exploratory analysis of both nuclear and mitochondrial genetic markers can be deemed essential for a clear demarcation between these closely related parasites. An additional genetic marker could not be included in the present study due to the non-availability of the sequences of the additional marker of the same field samples/isolates in the nucleotide databases, but this is a future prospective of this study. In addition, this analysis could also provide support for the existence of the genetic lineages in them.

The misidentification of a gene sequence of a parasite has a negative impact on the research community. The incorrectly annotated sequences of the 18S rRNA gene of *Theileria* spp. might be used by other researchers to identify the 18S rRNA sequences of new isolates or field samples, thus perpetuating the error. The results of the studies based on or involving misannotated sequences can be misleading. Therefore, the misidentification is to be avoided to avert its negative impact on the larger interests of researchers.

## Conclusion

In the course of analyzing the genetic diversity of *T. annulata*, we identified the nearly complete 18S rRNA gene sequences of other *Theileria* spp. that have not only been misidentified as *T. annulata* in the GenBank™, but are also published as *T. annulata*. Moreover, a high level of sequence conservation was noticed in the 18S rRNA gene of true *T. annulata* and *T. orientalis* sequences.

## Methods

### Data collection

Out of a total of 3168 nucleotide sequences of the 18S rRNA gene of *Theileria* spp. available in the NCBI database (https://www.ncbi.nlm.nih.gov/nuccore) up to March, 2022, all the sequences of *T. annulata* (*n* = 312) were downloaded. Smaller and truncated sequences were removed from the analysis; only sequences having nearly complete sequence length of the 18S rRNA gene (> 1527 bp) corresponding to positions one and 1527 of *T. annulata* Uttar Pradesh 1 isolate (MF287945; India) at the start and end, respectively, were used for further study. Similarly, at least one representative sequence of the same gene of different *Theileria* species infecting domestic and wild ruminants, viz., *Theileria lestoquardi*, *Theileria parva*, *Theileria taurotragi*, *Theileria ovis*, *Theileria cervi*, *Theileria capreoli*, *Theileria sinensis*, *Theileria buffeli*, *Theileria orientalis*, *Theileria luwenshuni*, *Theileria separata*, *Theileria uilenbergi*, *Theileria velifera* and *Theileria mutans*, was retrieved from the GenBank™. The details of all the sequences along with their accession numbers used in the current study are depicted in supplementary Table 1.

### Multiple sequence alignment and phylogenetic analysis based on the 18S rRNA gene

The sequences were aligned using multiple sequence alignment program MAFFT version 7 [42] and edited manually using BioEdit version 7.0.5.3 [43] as described by Nehra et al. [44], so that all the sequences started and ended at the homologous nucleotide positions. The multiple sequence alignments of the 18S rRNA gene sequences of different *Theileria* spp. were performed using MegAlign (DNASTAR) and BioEdit [43]. The nucleotide identities were computed using the ClustalW program of Lasergene 6.0 software [45].

Tamura-Nei model of maximum likelihood method was used for phylogenetic analysis and the best fit model of substitution was found to be TN93 + G + I [46]. The phylogenetic analysis of all nearly complete 18S rRNA sequences retrieved from the GenBank™ was performed using MEGA-X software version 10.1.7 [47]. The tree with the highest log likelihood (-6087.39) is shown. The percentage of trees in which the associated taxa clustered together is shown next to the branches. Initial tree(s) for the heuristic search were obtained automatically by applying Neighbor-Join and BioNJ algorithms to a matrix of pairwise distances estimated using the Maximum Composite Likelihood (MCL) approach, and then the topology was selected with superior log likelihood value. A discrete gamma distribution was applied with two categories (+ G, parameter = 0.16) and the rate variation model allowed 40.66% sites to be evolutionarily invariable. The tree was drawn to scale, with branch lengths measured in the number of substitutions per site. This analysis involved 87 nucleotide sequences with a total of 1564 positions in the final dataset. *Babesia duncani* (MH333111, USA) was used as an outgroup species for rooting (Fig. 1).

## Supplementary Information

**Additional file 1. Supplementary Table 1.** The details of *T. annulata* isolates/strains and *Theileria* species infecting domestic and wild ruminants originating from different countries used in sequence and phylogenetic analyses in the current study.

**Additional file 2. Supplementary Fig. 1.** Circular phylogram of different *T. annulata* isolates with the other *Theileria* species based on nearly complete 18S rRNA gene sequences.

**Additional file 3. Supplementary File 1: **Multiple sequence alignment of the nearly complete 18S rRNA gene sequences of true *T. annulata* isolates/ strains used in the sequence and phylogenetic analyses in the present study.

**Additional file 4. Supplementary File 2: **Multiple sequence alignment of the nearly complete 18S rRNA gene sequences of *T. orientalis *group used in the sequence and phylogenetic analyses in the present study.

## Data Availability

The datasets generated and/or analyzed during the current study are available in the GenBank™ repository (https://www.ncbi.nlm.nih.gov/) and the accession numbers are listed in supplementary Table 1.
