# Supplementary material for: An insight into misidentification of the small-subunit ribosomal RNA (18S rRNA) gene sequences of Theileria spp. as Theileria annulata
Source: BMC Vet Res. 2022 Dec 28;18:454. doi: 10.1186/s12917-022-03540-w (PMC9795727; doi:10.1186/s12917-022-03540-w)
Supplement: Supplementary file 1 — Additional file 1. Supplementary Table 1. The details of T. annulata isolates/strains and Theileria species infecting domestic and wild ruminants originating from different countries used in sequence and phylogenetic analyses in the current study. [file 12917_2022_3540_MOESM1_ESM.docx]

**Supplementary Table 1. The details of *T. annulata* isolates/strains and *Theileria* species infecting domestic and wild ruminants originating from different countries used in sequence and phylogenetic analyses in the current study**

| Place of sampling | Parasite | Country | Year of isolation | Host species | Isolate code | Sequence length (bp) | Position | Accession No. | Reference |
| --- | --- | --- | --- | --- | --- | --- | --- | --- | --- |
| Bareilly, Uttar Pradesh | *T. annulata* | India | 2018 | Adult cattle | Uttar Pradesh 1 | 1527 | 1-1527 | MF287945 | Kundave et al. 2019 |
| Bareilly, Uttar Pradesh | *T. annulata* | India | 2018 | Adult cattle | Uttar Pradesh 2 | 1528 | 1-1528 | MF287946 | Kundave et al. 2019 |
| Bareilly, Uttar Pradesh | *T. annulata* | India | 2018 | Adult cattle | Uttar Pradesh 3 | 1530 | 2-1530 | MF287947 | Kundave et al. 2019 |
| Udham Singh Nagar, Uttarakhand | *T. annulata* | India | 2018 | Adult cattle | Uttarakhand 1 | 1528 | 1-1528 | MF287941 | Kundave et al. 2019 |
| Udham Singh Nagar, Uttarakhand | *T. annulata* | India | 2018 | Adult cattle | Uttarakhand 2 | 1536 | 1-1536 | MF287942 | Kundave et al. 2019 |
| Udham Singh Nagar, Uttarakhand | *T. annulata* | India | 2018 | Adult cattle | Uttarakhand 3 | 1531 | 1-1531 | MF287943 | Kundave et al. 2019 |
| Udham Singh Nagar, Uttarakhand | *T. annulata* | India | 2018 | Adult cattle | Uttarakhand 4 | 1528 | 1-1528 | MF287944 | Kundave et al. 2019 |
| Bhubaneshwar, Orissa | *T. annulata* | India | 2018 | Adult cattle | Orissa 1 | 1529 | 1-1529 | MF287929 | Kundave et al. 2019 |
| Bhubaneshwar, Orissa | *T. annulata* | India | 2018 | Adult cattle | Orissa 2 | 1530 | 1-1530 | MF287930 | Kundave et al. 2019 |
| Bhubaneshwar, Orissa | *T. annulata* | India | 2018 | Adult cattle | Orissa 3 | 1530 | 1-1530 | MF287931 | Kundave et al. 2019 |
| Bhubaneshwar, Orissa | *T. annulata* | India | 2018 | Adult cattle | Orissa 4 | 1529 | 1-1529 | MF287932 | Kundave et al. 2019 |
| Anand, Gujarat | *T. annulata* | India | 2018 | Adult cattle | Gujarat 1 | 1532 | 1-1532 | MF287917 | Kundave et al. 2019 |
| Anand, Gujarat | *T. annulata* | India | 2018 | Adult cattle | Gujarat 2 | 1529 | 1-1529 | MF287918 | Kundave et al. 2019 |
| Anand, Gujarat | *T. annulata* | India | 2018 | Adult cattle | Gujarat 3 | 1538 | 1-1538 | MF287919 | Kundave et al. 2019 |
| Anand, Gujarat | *T. annulata* | India | 2018 | Adult cattle | Gujarat 4 | 1538 | 1-1538 | MF287920 | Kundave et al. 2019 |
| Hisar, Haryana | *T. annulata* | India | 2018 | Adult cattle | Haryana 1 | 1529 | 1-1529 | MF287921 | Kundave et al. 2019 |
| Hisar, Haryana | *T. annulata* | India | 2018 | Adult cattle | Haryana 2 | 1529 | 1-1529 | MF287922 | Kundave et al. 2019 |
| Hisar, Haryana | *T. annulata* | India | 2018 | Adult cattle | Haryana 4 | 1529 | 1-1529 | MF287924 | Kundave et al. 2019 |
| Ludhiana, Punjab | *T. annulata* | India | 2018 | Adult cattle | Punjab 1 | 1529 | 1-1529 | MF287933 | Kundave et al. 2019 |
| Ludhiana, Punjab | *T. annulata* | India | 2018 | Adult cattle | Punjab 2 | 1528 | 1-1528 | MF287934 | Kundave et al. 2019 |
| Ludhiana, Punjab | *T. annulata* | India | 2018 | Adult cattle | Punjab 3 | 1529 | 1-1529 | MF287935 | Kundave et al. 2019 |
| Ludhiana, Punjab | *T. annulata* | India | 2018 | Adult cattle | Punjab 4 | 1530 | 1-1530 | MF287936 | Kundave et al. 2019 |
| Akola, Maharashtra | *T. annulata* | India | 2018 | Adult cattle | Maharashtra 1 | 1528 | 1-1528 | MF287925 | Kundave et al. 2019 |
| Akola, Maharashtra | *T. annulata* | India | 2018 | Adult cattle | Maharashtra 3 | 1529 | 1-1529 | MF287927 | Kundave et al. 2019 |
| Akola, Maharashtra | *T. annulata* | India | 2018 | Adult cattle | Maharashtra 4 | 1529 | 1-1529 | MF287928 | Kundave et al. 2019 |
| Chennai, Tamil Nadu | *T. annulata* | India | 2018 | Adult cattle | Tamil Nadu 1 | 1536 | 1-1536 | MF287937 | Kundave et al. 2019 |
| Chennai, Tamil Nadu | *T. annulata* | India | 2018 | Adult cattle | Tamil Nadu 2 | 1529 | 1-1529 | MF287938 | Kundave et al. 2019 |
| Chennai, Tamil Nadu | *T. annulata* | India | 2018 | Adult cattle | Tamil Nadu 3 | 1536 | 1-1536 | MF287939 | Kundave et al. 2019 |
| Bhardhaman, West Bengal | *T. annulata* | India | 2018 | Adult cattle | West Bengal 1 | 1531 | 1-1531 | MF287949 | Kundave et al. 2019 |
| Bhardhaman, West Bengal | *T. annulata* | India | 2018 | Adult cattle | West Bengal 2 | 1536 | 1-1536 | MF287950 | Kundave et al. 2019 |
| Bhardhaman, West Bengal | *T. annulata* | India | 2018 | Adult cattle | West Bengal 3 | 1529 | 1-1529 | MF287951 | Kundave et al. 2019 |
| Bhardhaman, West Bengal | *T. annulata* | India | 2018 | Adult cattle | West Bengal 4 | 1529 | 1-1529 | MF287952 | Kundave et al. 2019 |
| Moradabad, Uttar Pradesh | *T. annulata* | India | 2016 | Adult cattle | MR | 1529 | 1-1529 | KT736498 | Shahzad M, Ram H, Kumar S, Chauhan RP, Sharma AK, Garg R, Goswami TK, Tiwari AK, Banerjee PS (Unpublished) |
| Bahraich, Uttar Pradesh | *T. annulata* | India | 2016 | Adult cattle | BJ | 1529 | 1-1529 | KT736497 | Shahzad M, Ram H, Kumar S, Chauhan RP, Sharma AK, Garg R, Goswami TK, Tiwari AK, Banerjee PS (Unpublished) |
| Bareilly, Uttar Pradesh | *T. annulata* | India | 2016 | Adult cattle | B1 | 1529 | 1-1529 | KT736496 | Shahzad M, Ram H, Kumar S, Chauhan RP, Sharma AK, Garg R, Goswami TK, Tiwari AK, Banerjee PS (Unpublished) |
| Muzaffarnagar, Uttar Pradesh | *T. annulata* | India | 2016 | Adult cattle | M1 | 1529 | 1-1529 | KT736495 | Shahzad M, Ram H, Kumar S, Chauhan RP, Sharma AK, Garg R, Goswami TK, Tiwari AK, Banerjee PS (Unpublished) |
| Manipur | *T. annulata* | India | 2016 | Adult cattle | NE | 1536 | 1-1536 | KT736499 | Shahzad M, Ram H, Kumar S, Chauhan RP, Sharma AK, Garg R, Goswami TK, Tiwari AK, Banerjee PS (Unpublished) |
| Krishna, Andhra Pradesh | *T. annulata* | India | 2015 | Cattle | TA1 | 1727 | 170-1698 | KT367866 | George et al. 2015 |
| Anantapur, Andhra Pradesh | *T. annulata* | India | 2015 | Cattle | TA2 | 1727 | 170-1698 | KT367867 | George et al. 2015 |
| Adilabad, Telangana | *T. annulata* | India | 2015 | Cattle | TA3 | 1727 | 170-1698 | KT367868 | George et al. 2015 |
| Medak, Telangana | *T. annulata* | India | 2015 | Cattle | TA4 | 1736 | 170-1707 | KT367869 | George et al. 2015 |
| Karimnagar, Telangana | *T. annulata* | India | 2015 | Cattle | TA5 | 1736 | 170-1707 | KT367870 | George et al. 2015 |
| Nizamabad, Telangana | *T. annulata* | India | 2015 | Cattle | TA6 | 1727 | 170-1698 | KT367871 | George et al. 2015 |
| Hyderabad, Telangana | *T. annulata* | India | 2015 | Cattle | TA7 | 1727 | 170-1698 | KT367872 | George et al. 2015 |
| Warangal, Telangana | *T. annulata* | India | 2015 | Cattle | TA8 | 1727 | 170-1698 | KT367873 | George et al. 2015 |
| Ranga Reddy, Telangana | *T. annulata* | India | 2015 | Cattle | TA9 | 1727 | 170-1698 | KT367874 | George et al. 2015 |
| Nallagonda, Telangana | *T. annulata* | India | 2015 | Cattle | TA10 | 1727 | 170-1698 | KT367875 | George et al. 2015 |
| Chittoor, Andhra Pradesh | *T. annulata* | India | 2015 | Cattle | TA11 | 1727 | 170-1698 | KT367876 | George et al. 2015 |
| Visakhapatnam, Andhra Pradesh | *T. annulata* | India | 2015 | Cattle | TA12 | 1727 | 170-1698 | KT367877 | George et al. 2015 |
| Srikakulam, Andhra Pradesh | *T. annulata* | India | 2015 | Cattle | TA13 | 1727 | 170-1698 | KT367878 | George et al. 2015 |
| Hisar | *T. annulata* | India | 1991 | Bovine | Not assigned | 1744 | 185-1712 | M64243 | Gajadhar et al. 1991 |
| Shanmenxia | *T. annulata* | China | 2013 | Cattle | Not assigned | 1741 | 184-1712 | KF559356 | Tian et al. 2013 |
| Neimeng | *T. annulata* | China | 2008 | Cattle | Not assigned | 1758 | 186-1714 | EU083801 | Gou et al. 2013 |
| Unknown | *T. annulata* | China | 2010 | Unknown | Not assigned | 1741 | 185-1713 | HM538216 | Wang L, Zhao J, Song Q, Xu L, Zhou Y (Unpublished) |
| Ningxia | *T. annulata* | China | 2008 | *Bos taurus* | Not assigned | 1758 | 186-1714 | EU083800 | Liu A, Yin H, Luo J (Unpublished) |
| Xinjiang, Yining | *T. annulata* | China | 2008 | *Bos taurus* | Yining | 1741 | 185-1713 | EU073963 | Liu AH, Yin H, Luo JX (Unpublished) |
| Xinjiang | *T. annulata* | China | 2008 | *Bos taurus* | Xinjiang | 1760 | 186-1716 | EU083799 | Liu A, Yin H, Luo J (Unpublished) |
| Alborz | *T. annulata* | Iran | 2012 | Cattle | Alb1 | 1977 | 339-1867 | KF429799 | Afshari A, Habibi GR, Esmaeilnia K, Shayan P (Unpublished) |
| Alborz | *T. annulata* | Iran | 2012 | Cattle | Alb2 | 1834 | 191-1719 | KF429800 | Afshari A, Habibi GR, Esmaeilnia K, Shayan P (Unpublished) |
| Alborz | *T. annulata* | Iran | 2012 | Cattle | Alb3 | 1853 | 215-1743 | KF429793 | Afshari A, Habibi GR, Esmaeilnia K, Shayan P (Unpublished) |
| Unknown | *T. annulata* | Iran | 2013 | Macroschizont infected macrophage/B cell line | Vaccine S15 | 1889 | 209-1737 | KF429795 | Afshari A, Habibi GR, Esmaeilnia K, Shayan P (Unpublished) |
| Elazig | *T. annulata* | Turkey | 2005 | Cattle | Not assigned | 1741 | 185-1713 | AY524666 | Aktas et al. 2007 |
| Elazig | *T. annulata* | Turkey | 2005 | Cattle | Turkey 1 | 1741 | 185-1713 | AY508462 | Aktas et al. 2007 |
| Malatya | *T. annulata* | Turkey | 2005 | Cattle | Turkey 5 | 1741 | 185-1713 | AY508466 | Aktas et al. 2007 |
| Malatya | *T. annulata* | Turkey | 2005 | Cattle | Turkey 5 | 1741 | 185-1713 | AY508465 | Aktas et al. 2007 |
| Malatya | *T. annulata* | Turkey | 2005 | Cattle | Turkey 5 | 1741 | 185-1713 | AY508467 | Aktas et al. 2007 |
| Malatya | *T. annulata* | Turkey | 2005 | Cattle | Turkey 7 | 1741 | 185-1713 | AY508471 | Aktas et al. 2007 |
| Malatya | *T. annulata* | Turkey | 2005 | Cattle | Turkey 7 | 1741 | 185-1713 | AY508472 | Aktas et al. 2007 |
| Sevilla, Andalousia | *T. annulata* | Spain | 2006 | Dog | Spain 2 | 1732 | 180-1708 | DQ287944 | Criado et al. 2006 |
| Unknown | *T. annulata* | Italy | 2009 | Cattle | ITC01 | 1732 | 180-1708 | FJ426369 | Criado-Fornelio et al. 2009 |
| Unknown | *T. lestoquardi* | China | 2000 | Sheep | Vaccine strain | 1745 | 185-1713 | AF081135 | Schnittger et al. 2000 |
| Lahr | *T. lestoquardi* | Iran | 2010 | Sheep | Lahr (Iran) | 1757 | 185-1713 | AJ006446 | Katzer F, McKellar S, Kirvar E, Shiels B (Unpublished) |
| Kiambu | *T. parva* | Kenya | 1993 | Cattle | Kiambu 4 | 1742 | 185-1714 | L02366 | Allsopp et al. 1993 |
| Vaalwater | *T. taurotragi* | South Africa | 1994 | Cattle | Vaalwater | 1737 | 185-1709 | L19082 | Allsopp et al. 1994 |
| Xinjiang | *T. ovis* | China | 2011 | Sheep | Xinjiang | 1764 | 185-1720 | FJ603460 | Li et al. 2011 |
| Wisconsin | *T. cervi* | USA | 2005 | Elk | Wisconsin elk 3 clone 16 | 1748 | 185-1720 | AY735129 | Bendele KG, Holman PJ (Unpublished) |
| Qilian-Mountain | *T. capreoli* | China | 2014 | Red deer | Qilian-Mountain 13 | 1751 | 185-1723 | KJ188219 | Li et al. 2014 |
| Unknown | *T. sinensis* | Malaysia | 2020 | Unknown | T22 | 1670 | 135-1665 | MT271902 | Agina OA, Isa M, Ajat M, Zamri-Saad M, Hamzah H (Unpublished) |
| Xiaogan | *T. buffeli* | China | 2012 | Water buffalo | Xiaogan17 | 1743 | 185-1715 | HQ840966 | He et al. 2012 |
| Hongan | *T. buffeli* | China | 2012 | Water buffalo | Hongan2 | 1744 | 185-1716 | HQ840964 | He et al. 2012 |
| Unknown | *T. buffeli* | India | 2006 | Unknown | Not assigned | 1744 | 185-1716 | EF126184 | Rengaraju T, Vanlalhmuaka, Bansal GC, Ray DD, Sankar M (Unpublished) |
| Kempsey | *T. orientalis* | Australia | 2011 | Cattle | Kempsey 6 | 1713 | 158-1685 | AB520953 | Kamau et al. 2011 |
| Forster | *T. orientalis* | Australia | 2011 | Cattle | Forster-4 | 1716 | 158-1688 | AB520954 | Kamau et al. 2011 |
| Clifton | *T. orientalis* | Australia | 2011 | Cattle | Clifton-41 | 1723 | 158-1695 | AB520956 | Kamau et al. 2011 |
| Unknown | *T. orientalis* | South Korea | 2020 | Cattle | C-GN-11 | 1695 | 150-1687 | MT889728 | Kwak and Seo 2020 |
| Unknown | *T. orientalis* | Bangladesh | 2017 | Cattle | BDH3 | 1659 | 106-1644 | MF576178 | Roy et al. 2018 |
| Dushan | *T. luwenshuni* | China | 2013 | Small ruminant | Dushan | 1745 | 185-1717 | KC735145 | Yuan Y, Li Y, Liu Z, Luo J, Yin H (Unpublished) |
| Unknown | *T. separata* | South Africa | 2004 | Sheep | GU 17 | 1761 | 186-1717 | AY260175 | Schnittger et al. 2004 |
| Unknown | *T. uilenbergi* | China | 2010 | Sheep | Li 2 | 1744 | 185-1716 | JF719835 | Gou et al. 2012 |
| Lugurni | *T. velifera* | Tanzania | 1999 | Cattle | Not assigned | 1746 | 185-1714 | AF097993 | Gubbels et al. 1999 |
| Intona | *T. mutans* | Kenya | 1999 | Bovine | Intona | 1737 | 185-1709 | AF078815 | Chae et al. 1999 |
| Washington | *Babesia duncani* | USA | 2018 | Human | WA1 | 1769 | 194-1738 | MH333111 | Swei et al. 2019 |
