# Supplementary material for: An insight into misidentification of the small-subunit ribosomal RNA (18S rRNA) gene sequences of Theileria spp. as Theileria annulata
Source: BMC Vet Res. 2022 Dec 28;18:454. doi: 10.1186/s12917-022-03540-w (PMC9795727; doi:10.1186/s12917-022-03540-w)

**Supplementary Fig. 1.** Circular phylogram of different *T. annulata* isolates with other *Theileria* species based on nearly complete 18S rRNA gene sequences.


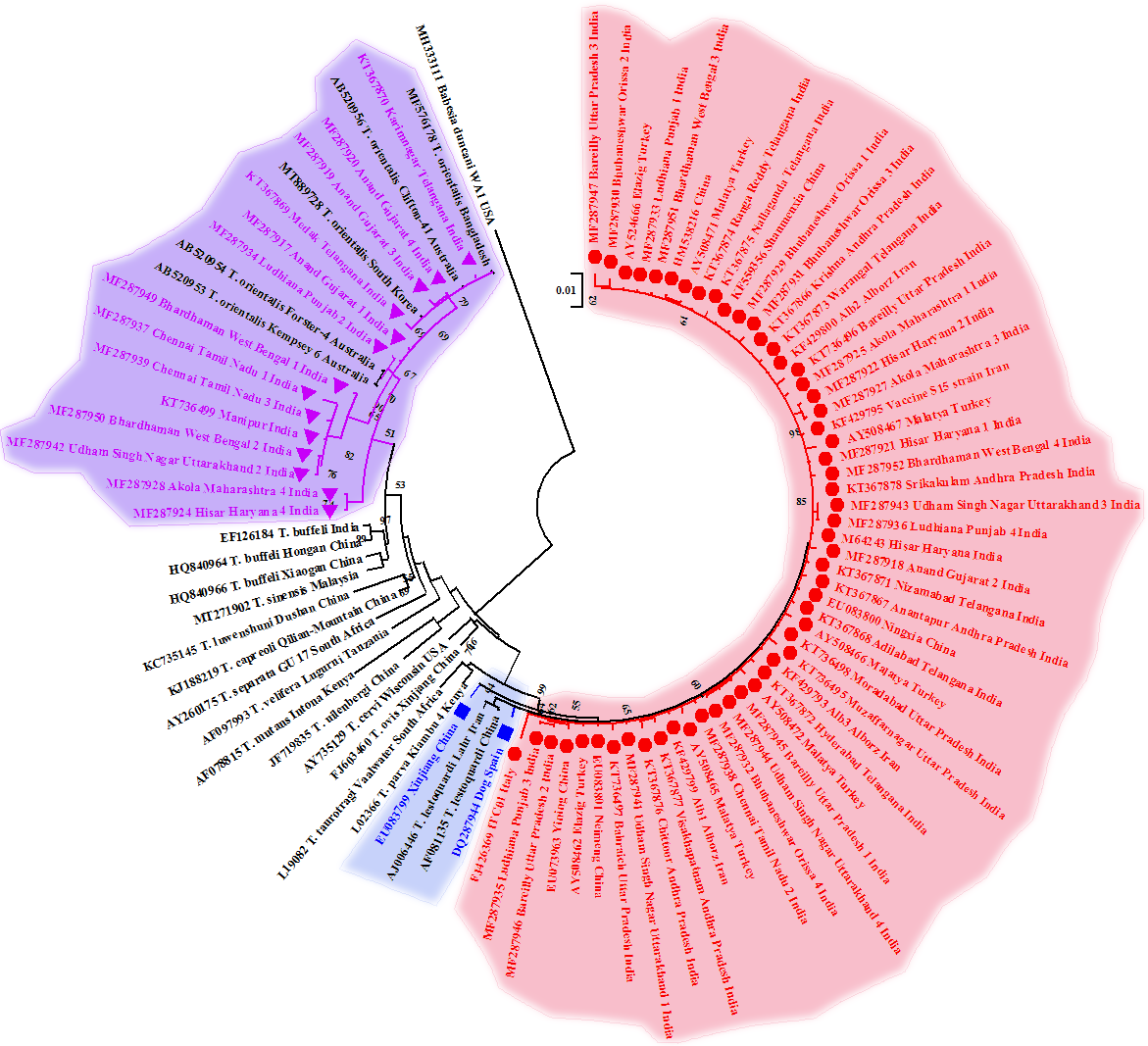

Supplement: Supplementary file 2 — Additional file 2. Supplementary Fig. 1. Circular phylogram of different T. annulata isolates with the other Theileria species based on nearly complete 18S rRNA gene sequences. [file 12917_2022_3540_MOESM2_ESM.docx]
