# Supplementary material for: An insight into misidentification of the small-subunit ribosomal RNA (18S rRNA) gene sequences of Theileria spp. as Theileria annulata
Source: BMC Vet Res. 2022 Dec 28;18:454. doi: 10.1186/s12917-022-03540-w (PMC9795727; doi:10.1186/s12917-022-03540-w)
Supplement: Supplementary file 3 — Additional file 3. Supplementary File 1: Multiple sequence alignment of the nearly complete 18S rRNA gene sequences of true T. annulata isolates/ strains used in the sequence and phylogenetic analyses in the present study. [file 12917_2022_3540_MOESM3_ESM.pdf]

```
    GGCGGCGTTTATTAGACCTAAAACCAAACCGCTTGCGGTGTCCGGTGATTCATAATAAATATGCGAATCGTACTCTGTACGATGTATCATTCAAGTTTCTGACCTATCAGCTTTGGACGG Majority
             10        20        30        40        50        60        70        80        90       100       110       120
1   ........................................................................................................................ AY508462_Elazig_Turkey
1   ........................................................................................................................ AY508465_Malatya_Turkey
1   ........................................................................................................................ AY508466_Malatya_Turkey
1   ........................................................................................................................ AY508467_Malatya_Turkey
1   ........................................................................................................................ AY508471_Malatya_Turkey
1   .................................................................................G...................................... AY508472_Malatya_Turkey
1   ........................................................................................................................ AY524666_Elazig_Turkey
1   ....................................................C.....................................................A............. EU073963_Yining_China
1   ........................................................................................................................ EU083800_Ningxia_China
1   ........................................................................................................................ EU083801_Neimeng_China
1   ............................................................T........................................................... FJ426369_ITC01_Italy
1   ........................................................................................................................ HM538216_China
1   ........................................................................................................................ KF429793_Alb3 Alborz_Iran
1   ........................................................................................................................ KF429795_Vaccine S15 strain_Iran
1   ........................................................................................................................ KF429799_Alb1 Alborz_Iran
1   ........................................................................................................................ KF429800_Alb2_Alborz_Iran
1   ........................................................................................................................ KF559356_Shanmenxia_China
1   ........................................................................................................................ KT367866_Krishna_Andhra Pradesh_India
1   ........................................................................................................................ KT367867_Anantapur_Andhra Pradesh_India
1   ........................................................................................................................ KT367868_Adilabad_Telangana_India
1   ........................................................................................................................ KT367871_Nizamabad_Telangana_India
1   ........................................................................................................................ KT367872_Hyderabad_Telangana_India
1   ........................................................................................................................ KT367873_Warangal_Telangana_India
1   ........................................................................................................................ KT367874_Ranga Reddy_Telangana_India
1   ........................................................................................................................ KT367875_Nallagonda_Telangana_India
1   ........................................................................................................................ KT367876_Chittoor_Andhra Pradesh_India
1   ...............................................................................................C........................ KT367877_Visakhapatnam_Andhra Pradesh_I
1   ........................................................................................................................ KT367878_Srikakulam_Andhra Pradesh_Indi
1   ........................................................................................................................ KT736495_Muzaffarnagar_Uttar Pradesh_In
1   ........................................................................................................................ KT736496_Bareilly_Uttar Pradesh_India
1   ........................................................................................................................ KT736497_Bahraich_Uttar Pradesh_India
1   ........................................................................................................................ KT736498_Moradabad_Uttar Pradesh_India
1   ........................................................................................................................ M64243_Hisar_Haryana_India
1   ........................................................................................................................ MF287918_Anand_Gujarat 2_India
1   ........................................................................................................................ MF287921_Hisar_Haryana 1_India
1   ........................................................................................................................ MF287922_Hisar_Haryana 2_India
1   ........................................................................................................................ MF287925_Akola_Maharashtra 1_India
1   ........................................................................................................................ MF287927_Akola_Maharashtra 3_India
1   ........................................................................................................................ MF287929_Bhubaneshwar_Orissa 1_India
1   ............................................................................................G...................C....... MF287930_Bhubaneshwar_Orissa 2_India
1   ........................................................................................................................ MF287931_Bhubaneshwar_Orissa 3_India
1   ........................................................................................................................ MF287932_Bhubaneshwar_Orissa 4_India
1   ........................................................................................................................ MF287933_Ludhiana_Punjab 1_India
1   ........................................................................................................................ MF287935_Ludhiana_Punjab 3_India
1   ........................................................................................................................ MF287936_Ludhiana_Punjab 4_India
1   ........................................................................................................................ MF287938_Chennai_Tamil Nadu 2_India
1   ........................................................................................................................ MF287941_Udham Singh Nagar_Uttarakhand
1   ........................................................................................................................ MF287943_Udham Singh Nagar_Uttarakhand
1   ........................................................................................................................ MF287944_Udham Singh Nagar_Uttarakhand
1   ........................................................................................................................ MF287945_Bareilly_Uttar Pradesh 1_India
1   ........................................................................................................................ MF287946_Bareilly_Uttar Pradesh 2_India
1   ........................................................................................................................ MF287947_Bareilly_Uttar Pradesh 3_India
1   ........................................................................................................................ MF287951_Bhardhaman_West Bengal 3_India
1   ........................................................................................................................ MF287952_Bhardhaman_West Bengal 4_India

    TAGGGTATTGGCCTACCGGGGCAACGACGGGTAACGGGGAATTAGGGTTCGATTCCGGAGAGGGAGCCTGAGAAACGGCTACCACATCTAAGGAAGGCAGCAGGCGCGCAAATTACCCAA Majority
            130       140       150       160       170       180       190       200       210       220       230       240
121 ........................................................................................................................ AY508462_Elazig_Turkey
121 ........................................................................................................................ AY508465_Malatya_Turkey
121 ........................................................................................................................ AY508466_Malatya_Turkey
121 ........................................................................................................................ AY508467_Malatya_Turkey
121 ........................................................................................................................ AY508471_Malatya_Turkey
121 ........................................................................................................................ AY508472_Malatya_Turkey
121 ........................................................................................................................ AY524666_Elazig_Turkey
121 ........................................................................................................................ EU073963_Yining_China
121 ........................................................................................................................ EU083800_Ningxia_China
121 ........................................................................................................................ EU083801_Neimeng_China
121 .......................G................................................................................................ FJ426369_ITC01_Italy
121 ........................................................................................................................ HM538216_China
121 ........................................................................................................................ KF429793_Alb3 Alborz_Iran
121 ........................................................................................................................ KF429795_Vaccine S15 strain_Iran
121 ........................................................................................................................ KF429799_Alb1 Alborz_Iran
121 ........................................................................................................................ KF429800_Alb2_Alborz_Iran
121 ........................................................................................................................ KF559356_Shanmenxia_China
121 ........................................................................................................................ KT367866_Krishna_Andhra Pradesh_India
121 ........................................................................................................................ KT367867_Anantapur_Andhra Pradesh_India
121 ........................................................................................................................ KT367868_Adilabad_Telangana_India
121 ........................................................................................................................ KT367871_Nizamabad_Telangana_India
121 ..........................................................................................G............................. KT367872_Hyderabad_Telangana_India
121 .......................................................................................................................G KT367873_Warangal_Telangana_India
121 ........................................................................................................................ KT367874_Ranga Reddy_Telangana_India
121 ..................................................T..................................................................... KT367875_Nallagonda_Telangana_India
121 ........................................................................................................................ KT367876_Chittoor_Andhra Pradesh_India
121 ........................................................................................................................ KT367877_Visakhapatnam_Andhra Pradesh_I
121 ........................................................................................................................ KT367878_Srikakulam_Andhra Pradesh_Indi
121 ........................................................................................................................ KT736495_Muzaffarnagar_Uttar Pradesh_In
121 ........................................................................................................................ KT736496_Bareilly_Uttar Pradesh_India
121 ........................................................................................................................ KT736497_Bahraich_Uttar Pradesh_India
121 ........................................................................................................................ KT736498_Moradabad_Uttar Pradesh_India
121 ........................................................................................................................ M64243_Hisar_Haryana_India
121 ........................................................................................................................ MF287918_Anand_Gujarat 2_India
121 ........................................................................................................................ MF287921_Hisar_Haryana 1_India
121 ........................................................................................................................ MF287922_Hisar_Haryana 2_India
121 ........................................................................................................................ MF287925_Akola_Maharashtra 1_India
121 ........................................................................................................................ MF287927_Akola_Maharashtra 3_India
121 ........................................................................................................................ MF287929_Bhubaneshwar_Orissa 1_India
121 ..................................................................................................................T..... MF287930_Bhubaneshwar_Orissa 2_India
121 ........................................................................................................................ MF287931_Bhubaneshwar_Orissa 3_India
121 ........................................................A............................................................... MF287932_Bhubaneshwar_Orissa 4_India
121 ........................................................................................................................ MF287933_Ludhiana_Punjab 1_India
121 ........................................................................................................................ MF287935_Ludhiana_Punjab 3_India
121 ........................................................................................................................ MF287936_Ludhiana_Punjab 4_India
121 ........................................................................................................................ MF287938_Chennai_Tamil Nadu 2_India
121 ........................................................................................................................ MF287941_Udham Singh Nagar_Uttarakhand
121 ........................................................................................................................ MF287943_Udham Singh Nagar_Uttarakhand
121 ........................................................A............................................................... MF287944_Udham Singh Nagar_Uttarakhand
121 ........................................................A............................................................... MF287945_Bareilly_Uttar Pradesh 1_India
121 ........................................................................................................................ MF287946_Bareilly_Uttar Pradesh 2_India
121 ........................................................................................................................ MF287947_Bareilly_Uttar Pradesh 3_India
121 ........................................................................................................................ MF287951_Bhardhaman_West Bengal 3_India
121 ........................................................................................................................ MF287952_Bhardhaman_West Bengal 4_India
```

```
    TCCTGACACAGGGAGGTAGTGACAAGAAATAACAATACGGGGCTTAAAGTCTTGTAATTGGAATGATGGGAATTTAAACCTCTTCCAGAGTATCAATTGGAGGGCAAGTCTGGTGCCAGC Majority
             250       260       270       280       290       300       310       320       330       340       350       360
241 ........................................................................................................................ AY508462_Elazig_Turkey
241 ............................G........................................................................................... AY508465_Malatya_Turkey
241 ........................................................................................................................ AY508466_Malatya_Turkey
241 ........................................................................................................................ AY508467_Malatya_Turkey
241 ........................................................................................................................ AY508471_Malatya_Turkey
241 ........................................................................................................................ AY508472_Malatya_Turkey
241 ........................................................................................................................ AY524666_Elazig_Turkey
241 ........................................................................................................................ EU073963_Yining_China
241 ........................................................................................................................ EU083800_Ningxia_China
241 ........................................................................................................................ EU083801_Neimeng_China
241 ........................................................................................................................ FJ426369_ITC01_Italy
241 ........................................................................................................................ HM538216_China
241 ........................................................................................................................ KF429793_Alb3 Alborz_Iran
241 ........................................................................................................................ KF429795_Vaccine S15 strain_Iran
241 ........................................................................................................................ KF429799_Alb1 Alborz_Iran
241 ........................................................................................................................ KF429800_Alb2_Alborz_Iran
241 ........................................................................................................................ KF559356_Shanmenxia_China
241 ........................................................................................................................ KT367866_Krishna_Andhra Pradesh_India
241 ........................................................................................................................ KT367867_Anantapur_Andhra Pradesh_India
241 ........................................................................................................................ KT367868_Adilabad_Telangana_India
241 ........................................................................................................................ KT367871_Nizamabad_Telangana_India
241 ........................................................................................................................ KT367872_Hyderabad_Telangana_India
241 ........................................................................................................................ KT367873_Warangal_Telangana_India
241 ........................................................................................................................ KT367874_Ranga Reddy_Telangana_India
241 ........................................................................................................................ KT367875_Nallagonda_Telangana_India
241 ........................................................................................................................ KT367876_Chittoor_Andhra Pradesh_India
241 ....................A................................................................................................... KT367877_Visakhapatnam_Andhra Pradesh_I
241 ........................................................................................................................ KT367878_Srikakulam_Andhra Pradesh_Indi
241 ........................................................................................................................ KT736495_Muzaffarnagar_Uttar Pradesh_In
241 ........................................................................................................................ KT736496_Bareilly_Uttar Pradesh_India
241 ........................................................................................................................ KT736497_Bahraich_Uttar Pradesh_India
241 ........................................................................................................................ KT736498_Moradabad_Uttar Pradesh_India
241 ........................................................................................................................ M64243_Hisar_Haryana_India
241 ........................................................................................................................ MF287918_Anand_Gujarat 2_India
241 ........................................................................................................................ MF287921_Hisar_Haryana 1_India
241 .........................................................................................A.............................. MF287922_Hisar_Haryana 2_India
241 .........................................-.............................................................................. MF287925_Akola_Maharashtra 1_India
241 .........................................................................................A.............................. MF287927_Akola_Maharashtra 3_India
241 ........................................................................................................................ MF287929_Bhubaneshwar_Orissa 1_India
241 ........................................................................................................................ MF287930_Bhubaneshwar_Orissa 2_India
241 ........................................................................................................................ MF287931_Bhubaneshwar_Orissa 3_India
241 ........................................................................................................................ MF287932_Bhubaneshwar_Orissa 4_India
241 ........................................................................................................................ MF287933_Ludhiana_Punjab 1_India
241 ........................................................................................................................ MF287935_Ludhiana_Punjab 3_India
241 ........................................................................................................................ MF287936_Ludhiana_Punjab 4_India
241 ........................................................................................................................ MF287938_Chennai_Tamil Nadu 2_India
241 ........................................................................................................................ MF287941_Udham Singh Nagar_Uttarakhand
241 ........................................................................................................................ MF287943_Udham Singh Nagar_Uttarakhand
241 ........................................................................................................................ MF287944_Udham Singh Nagar_Uttarakhand
241 ........................................................................................................................ MF287945_Bareilly_Uttar Pradesh 1_India
241 ........................................................................................................................ MF287946_Bareilly_Uttar Pradesh 2_India
241 ........................................................................................................................ MF287947_Bareilly_Uttar Pradesh 3_India
241 ..................................................................................C..................................... MF287951_Bhardhaman_West Bengal 3_India
241 ........................................................................................................................ MF287952_Bhardhaman_West Bengal 4_India

    AGCCGCGGTAATTCCAGCTCCAATAGCGTATATTAAAATTGTTGCAGTTAAAAAGCTCGTAGTTGAATTTCTGCTGCATTGCTTGTGTCCCTCTGGGGTCTGTGCATGTGGCTTTTTTCG Majority
             370       380       390       400       410       420       430       440       450       460       470       480
361 ........................................................................................................................ AY508462_Elazig_Turkey
361 ........................................................................................................................ AY508465_Malatya_Turkey
361 ........C.......................................................................................................C....... AY508466_Malatya_Turkey
361 ..........................................C............................................................................. AY508467_Malatya_Turkey
361 ........................................................................................................................ AY508471_Malatya_Turkey
361 ........................................................................................................................ AY508472_Malatya_Turkey
361 ........................................................................................................................ AY524666_Elazig_Turkey
361 ........................................................................................T............................... EU073963_Yining_China
361 ........................................................................................................................ EU083800_Ningxia_China
361 ........................................................................................................................ EU083801_Neimeng_China
361 ........................................................................................................................ FJ426369_ITC01_Italy
361 ........................................................................................................................ HM538216_China
361 ........................................................................................................................ KF429793_Alb3 Alborz_Iran
361 ........................................................................................................................ KF429795_Vaccine S15 strain_Iran
361 ........................................................................................................................ KF429799_Alb1 Alborz_Iran
361 ........................................................................................................................ KF429800_Alb2_Alborz_Iran
361 ........................................................................................................................ KF559356_Shanmenxia_China
361 ........................................................................................................................ KT367866_Krishna_Andhra Pradesh_India
361 ........................................................................................................................ KT367867_Anantapur_Andhra Pradesh_India
361 ........................................................................................................................ KT367868_Adilabad_Telangana_India
361 ........................................................................................................................ KT367871_Nizamabad_Telangana_India
361 ........................................................................................................................ KT367872_Hyderabad_Telangana_India
361 ........................................................................................................................ KT367873_Warangal_Telangana_India
361 ........................................................................................................................ KT367874_Ranga Reddy_Telangana_India
361 ........................................................................................................................ KT367875_Nallagonda_Telangana_India
361 ........................................................................................................................ KT367876_Chittoor_Andhra Pradesh_India
361 ........................................................................................................................ KT367877_Visakhapatnam_Andhra Pradesh_I
361 ........................................................................................................................ KT367878_Srikakulam_Andhra Pradesh_Indi
361 ........................................................................................................................ KT736495_Muzaffarnagar_Uttar Pradesh_In
361 ..T.....................................................A............................................................... KT736496_Bareilly_Uttar Pradesh_India
361 ........................................................................................................................ KT736497_Bahraich_Uttar Pradesh_India
361 ........................................................................................................................ KT736498_Moradabad_Uttar Pradesh_India
361 ........................................................................................................................ M64243_Hisar_Haryana_India
361 ........................................................................................................................ MF287918_Anand_Gujarat 2_India
361 ........................................................................................................................ MF287921_Hisar_Haryana 1_India
361 ........................................................................................................................ MF287922_Hisar_Haryana 2_India
360 ........................................................C............................................................... MF287925_Akola_Maharashtra 1_India
361 ........................................................................................................................ MF287927_Akola_Maharashtra 3_India
361 ........................................................................................................................ MF287929_Bhubaneshwar_Orissa 1_India
361 ........................................................................................................................ MF287930_Bhubaneshwar_Orissa 2_India
361 ........................................................................................................................ MF287931_Bhubaneshwar_Orissa 3_India
361 ........................................................................................................................ MF287932_Bhubaneshwar_Orissa 4_India
361 ........................................................................................................................ MF287933_Ludhiana_Punjab 1_India
361 ........................................................................................T............................... MF287935_Ludhiana_Punjab 3_India
361 ........................................................................................................................ MF287936_Ludhiana_Punjab 4_India
361 ........................................................................................................................ MF287938_Chennai_Tamil Nadu 2_India
361 ........................................................................................................................ MF287941_Udham Singh Nagar_Uttarakhand
361 ........................................................................................................................ MF287943_Udham Singh Nagar_Uttarakhand
361 ........................................................................................................................ MF287944_Udham Singh Nagar_Uttarakhand
361 ........................................................................................................................ MF287945_Bareilly_Uttar Pradesh 1_India
361 ........................................................................................T............................... MF287946_Bareilly_Uttar Pradesh 2_India
361 ........................................................................................................................ MF287947_Bareilly_Uttar Pradesh 3_India
361 ........................................................................................................................ MF287951_Bhardhaman_West Bengal 3_India
361 ........................................................................................................................ MF287952_Bhardhaman_West Bengal 4_India
```

```
    GACGGAGTTTCTTTGTCTGAATGTTTACTTTGAGAAAATTAGAGTGCTCAAAGCAGGCTTTCGCCTTGAATAGTTTAGCATGGAATAATAAAGTAGGACTTTGGTTCTATTTTGTTGGTT Majority
             490       500       510       520       530       540       550       560       570       580       590       600
481 ........................................................................................................................ AY508462_Elazig_Turkey
481 ........................................................................................................................ AY508465_Malatya_Turkey
481 ........................................................................................................................ AY508466_Malatya_Turkey
481 ..............................................................................................G......................... AY508467_Malatya_Turkey
481 ........................................................................................................................ AY508471_Malatya_Turkey
481 ........................................................................................................................ AY508472_Malatya_Turkey
481 ........................................................................................................................ AY524666_Elazig_Turkey
481 ........................................................................................................................ EU073963_Yining_China
481 ........................................................................................................................ EU083800_Ningxia_China
481 ........................................................................................................................ EU083801_Neimeng_China
481 ........................................................................................................................ FJ426369_ITC01_Italy
481 ...............................................................................................T........................ HM538216_China
481 ........................................................................................................................ KF429793_Alb3 Alborz_Iran
481 ........................................................................................................................ KF429795_Vaccine S15 strain_Iran
481 ........................................................................................................................ KF429799_Alb1 Alborz_Iran
481 ........................................................................................................................ KF429800_Alb2_Alborz_Iran
481 ........................................................................................................................ KF559356_Shanmenxia_China
481 ........................................................................................................................ KT367866_Krishna_Andhra Pradesh_India
481 ........................................................................................................................ KT367867_Anantapur_Andhra Pradesh_India
481 ........................................................................................................................ KT367868_Adilabad_Telangana_India
481 ........................................................................................................................ KT367871_Nizamabad_Telangana_India
481 ........................................................................................................................ KT367872_Hyderabad_Telangana_India
481 ........................................................................................................................ KT367873_Warangal_Telangana_India
481 ........................................................................................................................ KT367874_Ranga Reddy_Telangana_India
481 ........................................................................................................................ KT367875_Nallagonda_Telangana_India
481 ........................................................................................................................ KT367876_Chittoor_Andhra Pradesh_India
481 ........................................................................................................................ KT367877_Visakhapatnam_Andhra Pradesh_I
481 ........................................................................................................................ KT367878_Srikakulam_Andhra Pradesh_Indi
481 ........................................................................................................................ KT736495_Muzaffarnagar_Uttar Pradesh_In
481 ........................................................................................................................ KT736496_Bareilly_Uttar Pradesh_India
481 .G...................................................................................................................... KT736497_Bahraich_Uttar Pradesh_India
481 ........................................................................................................................ KT736498_Moradabad_Uttar Pradesh_India
481 ........................................................................................................................ M64243_Hisar_Haryana_India
481 ........................................................................................................................ MF287918_Anand_Gujarat 2_India
481 ........................................................................................................................ MF287921_Hisar_Haryana 1_India
481 ........................................................................................................................ MF287922_Hisar_Haryana 2_India
480 ........................................................................................................................ MF287925_Akola_Maharashtra 1_India
481 ........................................................................................................................ MF287927_Akola_Maharashtra 3_India
481 .................................................................................................G...................... MF287929_Bhubaneshwar_Orissa 1_India
481 ........................................................................................................................ MF287930_Bhubaneshwar_Orissa 2_India
481 ........................................................................................................................ MF287931_Bhubaneshwar_Orissa 3_India
481 ........................................................................................................................ MF287932_Bhubaneshwar_Orissa 4_India
481 ........................................................................................................................ MF287933_Ludhiana_Punjab 1_India
481 ........................................................................................................................ MF287935_Ludhiana_Punjab 3_India
481 ........................................G................................C.............................................. MF287936_Ludhiana_Punjab 4_India
481 ........................................................................................................................ MF287938_Chennai_Tamil Nadu 2_India
481 ........................................................................................................................ MF287941_Udham Singh Nagar_Uttarakhand
481 ........................................G................................C.............................................. MF287943_Udham Singh Nagar_Uttarakhand
481 ........................................................................................................................ MF287944_Udham Singh Nagar_Uttarakhand
481 ........................................................................................................................ MF287945_Bareilly_Uttar Pradesh 1_India
481 ........................................................................................................................ MF287946_Bareilly_Uttar Pradesh 2_India
481 .................................................................A.......................................C.............. MF287947_Bareilly_Uttar Pradesh 3_India
481 ........................................................................................................................ MF287951_Bhardhaman_West Bengal 3_India
481 ........................................................................................................................ MF287952_Bhardhaman_West Bengal 4_India

    TTAGGTACCAAAGTAATGGTTAATAGGAACAGTTGGGGG-CATTCGTATTTAACTGTCAGAGGTGAAATTCTTAGATTTGTTAAAGACGAACTACTGCGAAAGCATTTGCCAAGGATGTT Majority
             610       620       630       640       650       660       670       680       690       700       710       720
601 .......................................-................................................................................ AY508462_Elazig_Turkey
601 .......................................-................................................................................ AY508465_Malatya_Turkey
601 .......................................-................................................................................ AY508466_Malatya_Turkey
601 .......................................-................................................................................ AY508467_Malatya_Turkey
601 .......................................-................................................................................ AY508471_Malatya_Turkey
601 .......................................-................................................................................ AY508472_Malatya_Turkey
601 .......................................-................................................................................ AY524666_Elazig_Turkey
601 .......................................-................................................................................ EU073963_Yining_China
601 .......................................-................................................................................ EU083800_Ningxia_China
601 .......................................-...............................................M................................ EU083801_Neimeng_China
601 .......................................-................................................................................ FJ426369_ITC01_Italy
601 .......................................-................................................................................ HM538216_China
601 .......................................-................................................................................ KF429793_Alb3 Alborz_Iran
601 .......................................-................................................................................ KF429795_Vaccine S15 strain_Iran
601 .......................................-................................................................................ KF429799_Alb1 Alborz_Iran
601 .......................................-................................................................................ KF429800_Alb2_Alborz_Iran
601 .......................................-................................................................................ KF559356_Shanmenxia_China
601 .......................................-................................................................................ KT367866_Krishna_Andhra Pradesh_India
601 .......................................-..........C..................................................................... KT367867_Anantapur_Andhra Pradesh_India
601 .......................................-................................................................................ KT367868_Adilabad_Telangana_India
601 .......................................-................................................................................ KT367871_Nizamabad_Telangana_India
601 .......................................-................................................................................ KT367872_Hyderabad_Telangana_India
601 .......................................-................................................................................ KT367873_Warangal_Telangana_India
601 .......................................-..................G............................................................. KT367874_Ranga Reddy_Telangana_India
601 .......................................-...............................C................................................ KT367875_Nallagonda_Telangana_India
601 .......................................-................................................................................ KT367876_Chittoor_Andhra Pradesh_India
601 .......................................-................................................................................ KT367877_Visakhapatnam_Andhra Pradesh_I
601 .......................................-................................................................................ KT367878_Srikakulam_Andhra Pradesh_Indi
601 .......................................-................................................................................ KT736495_Muzaffarnagar_Uttar Pradesh_In
601 .......................................-................................................................................ KT736496_Bareilly_Uttar Pradesh_India
601 .......................................-................................................................................ KT736497_Bahraich_Uttar Pradesh_India
601 .......................................-................................................................................ KT736498_Moradabad_Uttar Pradesh_India
601 .......................................-..........................R..................................................... M64243_Hisar_Haryana_India
601 ............A..........................-............................................G................................... MF287918_Anand_Gujarat 2_India
601 .......................................-................................................................................ MF287921_Hisar_Haryana 1_India
601 .......................................-..........................................................................A..... MF287922_Hisar_Haryana 2_India
600 .......................................-................................................................................ MF287925_Akola_Maharashtra 1_India
601 .......................................-..........................................................................A..... MF287927_Akola_Maharashtra 3_India
601 .......................................-................................................................................ MF287929_Bhubaneshwar_Orissa 1_India
601 .......................................-................................................................................ MF287930_Bhubaneshwar_Orissa 2_India
601 ......................................A-................................................................................ MF287931_Bhubaneshwar_Orissa 3_India
601 .......................................-................................................................................ MF287932_Bhubaneshwar_Orissa 4_India
601 .......................................-................................................................................ MF287933_Ludhiana_Punjab 1_India
601 .......................................-................................................................................ MF287935_Ludhiana_Punjab 3_India
601 .......................................G................................................................................ MF287936_Ludhiana_Punjab 4_India
601 .......................................-................................................................................ MF287938_Chennai_Tamil Nadu 2_India
601 .......................................-................................................................................ MF287941_Udham Singh Nagar_Uttarakhand
601 .......................................-................................................................................ MF287943_Udham Singh Nagar_Uttarakhand
601 .......................................-................................................................................ MF287944_Udham Singh Nagar_Uttarakhand
601 .......................................-................................................................................ MF287945_Bareilly_Uttar Pradesh 1_India
601 .......................................-................................................................................ MF287946_Bareilly_Uttar Pradesh 2_India
601 .......................................-................................................................................ MF287947_Bareilly_Uttar Pradesh 3_India
601 .......................................-................................................................................ MF287951_Bhardhaman_West Bengal 3_India
601 ........T..............................-..................................................G............................. MF287952_Bhardhaman_West Bengal 4_India
```

```
    TTCATTAATCAAGAACGAAAGTTAGGGGATCGAAGACGATCAGATACCGTCGTAGTCCTAACCATAAACTATGCCGACTAGAGATTGGAGGTCGTCAGTTTTTACGACTCCTTCAGCACC Majority
             730       740       750       760       770       780       790       800       810       820       830       840
720 ........................................................................................................................ AY508462_Elazig_Turkey
720 ........................................................................................................................ AY508465_Malatya_Turkey
720 ......................................................................................................................T. AY508466_Malatya_Turkey
720 ........................................................................................................................ AY508467_Malatya_Turkey
720 ........................................................................................................................ AY508471_Malatya_Turkey
720 ........................................................................................................................ AY508472_Malatya_Turkey
720 ........................................................................................................................ AY524666_Elazig_Turkey
720 ........................................................................................................................ EU073963_Yining_China
720 ........................................................................................................................ EU083800_Ningxia_China
720 ........................................................................................................................ EU083801_Neimeng_China
720 ...T..................................................C................................C......GG.........T......C.C..... FJ426369_ITC01_Italy
720 ........................................................................................................................ HM538216_China
720 ........................................................................................................................ KF429793_Alb3 Alborz_Iran
720 ........................................................................................................................ KF429795_Vaccine S15 strain_Iran
720 ........................................................................................................................ KF429799_Alb1 Alborz_Iran
720 ........................................................................................................................ KF429800_Alb2_Alborz_Iran
720 ........................................................................................................................ KF559356_Shanmenxia_China
720 ........................................................................................................................ KT367866_Krishna_Andhra Pradesh_India
720 ........................................................................................................................ KT367867_Anantapur_Andhra Pradesh_India
720 ........................................................................................................................ KT367868_Adilabad_Telangana_India
720 ........................................................................................................................ KT367871_Nizamabad_Telangana_India
720 ........................................................................................................................ KT367872_Hyderabad_Telangana_India
720 ........................................................................................................................ KT367873_Warangal_Telangana_India
720 ........................................................................................................................ KT367874_Ranga Reddy_Telangana_India
720 .............................................................................................A.......................... KT367875_Nallagonda_Telangana_India
720 ........................................................................................................................ KT367876_Chittoor_Andhra Pradesh_India
720 ........................................................................................................................ KT367877_Visakhapatnam_Andhra Pradesh_I
720 ........................................................................................................................ KT367878_Srikakulam_Andhra Pradesh_Indi
720 C....................................................................................................................... KT736495_Muzaffarnagar_Uttar Pradesh_In
720 ........................................................................................................................ KT736496_Bareilly_Uttar Pradesh_India
720 ........................................................................................................................ KT736497_Bahraich_Uttar Pradesh_India
720 ........................................................................................................................ KT736498_Moradabad_Uttar Pradesh_India
720 ........................................................................................................................ M64243_Hisar_Haryana_India
720 ........................................................................................................................ MF287918_Anand_Gujarat 2_India
720 ........................................................................................................................ MF287921_Hisar_Haryana 1_India
720 ........................................................................................................................ MF287922_Hisar_Haryana 2_India
719 ........................................................................................................................ MF287925_Akola_Maharashtra 1_India
720 ........................................................................................................................ MF287927_Akola_Maharashtra 3_India
720 ........................................................................................................................ MF287929_Bhubaneshwar_Orissa 1_India
720 ........................................................................................................................ MF287930_Bhubaneshwar_Orissa 2_India
720 ........................................................................................................................ MF287931_Bhubaneshwar_Orissa 3_India
720 ........................................................................................................................ MF287932_Bhubaneshwar_Orissa 4_India
720 ........................................................................................................................ MF287933_Ludhiana_Punjab 1_India
720 ........................................................................................................................ MF287935_Ludhiana_Punjab 3_India
721 ........................................................................................................................ MF287936_Ludhiana_Punjab 4_India
720 ........................................................................................................................ MF287938_Chennai_Tamil Nadu 2_India
720 ........................................................................................................................ MF287941_Udham Singh Nagar_Uttarakhand
720 ........................................................................................................................ MF287943_Udham Singh Nagar_Uttarakhand
720 ........................................................................................................................ MF287944_Udham Singh Nagar_Uttarakhand
720 ........................................................................................................................ MF287945_Bareilly_Uttar Pradesh 1_India
720 ........................................................................................................................ MF287946_Bareilly_Uttar Pradesh 2_India
720 ........................................................................................................................ MF287947_Bareilly_Uttar Pradesh 3_India
720 ........................................................................................................................ MF287951_Bhardhaman_West Bengal 3_India
720 ........................................................................................................................ MF287952_Bhardhaman_West Bengal 4_India

    TTGAGAGAAATCAAAGTCTTTGGGTTCTGGGGGGAGTATGGTCGCAAGGCTGAAACTTAAAGGAATTGACGGAAGGGCACCACCAGG-CGTGGAGCCTGCGGCTTAATTTGACTCAACAC Majority
             850       860       870       880       890       900       910       920       930       940       950       960
840 .......................................................................................-................................ AY508462_Elazig_Turkey
840 .......................................................................................-................................ AY508465_Malatya_Turkey
840 .......................................................................................-................................ AY508466_Malatya_Turkey
840 .......................................................................................-................................ AY508467_Malatya_Turkey
840 .......................................................................................-................................ AY508471_Malatya_Turkey
840 .......................................................................................-................................ AY508472_Malatya_Turkey
840 .......................................................................................-................................ AY524666_Elazig_Turkey
840 .......................................................................................-................................ EU073963_Yining_China
840 .......................................................................................-................................ EU083800_Ningxia_China
840 .......................................................................................-................................ EU083801_Neimeng_China
840 C......................................................................................-................................ FJ426369_ITC01_Italy
840 .......................................................................................-................................ HM538216_China
840 .......................................................................................-................................ KF429793_Alb3 Alborz_Iran
840 .......................................................................................-................................ KF429795_Vaccine S15 strain_Iran
840 .......................................................................................-................................ KF429799_Alb1 Alborz_Iran
840 .......................................................................................-................................ KF429800_Alb2_Alborz_Iran
840 .......................................................................................-................................ KF559356_Shanmenxia_China
840 .......................................................................................-................................ KT367866_Krishna_Andhra Pradesh_India
840 .......................................................................................-................................ KT367867_Anantapur_Andhra Pradesh_India
840 .......................................................................................-................................ KT367868_Adilabad_Telangana_India
840 .......................................................................................-................................ KT367871_Nizamabad_Telangana_India
840 .......................................................................................-................................ KT367872_Hyderabad_Telangana_India
840 .......................................................................................-................................ KT367873_Warangal_Telangana_India
840 .......................................................................................-................................ KT367874_Ranga Reddy_Telangana_India
840 .......................................................................................-................................ KT367875_Nallagonda_Telangana_India
840 .......................................................................................-................................ KT367876_Chittoor_Andhra Pradesh_India
840 .......................................................................................-................................ KT367877_Visakhapatnam_Andhra Pradesh_I
840 .......................................................................................-................................ KT367878_Srikakulam_Andhra Pradesh_Indi
840 .......................................................................................-................................ KT736495_Muzaffarnagar_Uttar Pradesh_In
840 .......................................................................................-................................ KT736496_Bareilly_Uttar Pradesh_India
840 .......................................................................................-................................ KT736497_Bahraich_Uttar Pradesh_India
840 .......................................................................................-................................ KT736498_Moradabad_Uttar Pradesh_India
840 .......................................................................................-........-....................... M64243_Hisar_Haryana_India
840 .......................................................................................-................................ MF287918_Anand_Gujarat 2_India
840 .......................................................................................-................................ MF287921_Hisar_Haryana 1_India
840 .......................................................................................-................................ MF287922_Hisar_Haryana 2_India
839 .......................................................................................-................................ MF287925_Akola_Maharashtra 1_India
840 .......................................................................................-................................ MF287927_Akola_Maharashtra 3_India
840 .......................................................................................-................................ MF287929_Bhubaneshwar_Orissa 1_India
840 .......................................................................................G................................ MF287930_Bhubaneshwar_Orissa 2_India
840 .......................................................................................G................................ MF287931_Bhubaneshwar_Orissa 3_India
840 .......................................................................................-................................ MF287932_Bhubaneshwar_Orissa 4_India
840 .......................................................................................-................................ MF287933_Ludhiana_Punjab 1_India
840 .......................................................................................-................................ MF287935_Ludhiana_Punjab 3_India
841 .......................................................................................-................................ MF287936_Ludhiana_Punjab 4_India
840 .......................................................................................-................................ MF287938_Chennai_Tamil Nadu 2_India
840 .......................................................................................-........-....................... MF287941_Udham Singh Nagar_Uttarakhand
840 .......................................................................................-................................ MF287943_Udham Singh Nagar_Uttarakhand
840 .......................................................................................-........-....................... MF287944_Udham Singh Nagar_Uttarakhand
840 .......................................................................................-........-............-.......... MF287945_Bareilly_Uttar Pradesh 1_India
840 .......................................................................................-........-....................... MF287946_Bareilly_Uttar Pradesh 2_India
840 .......................................................................................-........G....................... MF287947_Bareilly_Uttar Pradesh 3_India
840 .......................................................................................-................................ MF287951_Bhardhaman_West Bengal 3_India
840 .......................................................................................-................................ MF287952_Bhardhaman_West Bengal 4_India
```

```
     GGGGAAACTCACCAGGTCCAGACAAAGGAAGGATTGACAGATTGATAGCTCTTT-CTTGATTCTTTGGGTGGTGGTGCATGGCCGTT-CTTAGTTGGTGGAGTGATTTGTCTGGTTAATT  Majority
              970       980       990      1000      1010      1020      1030      1040      1050      1060      1070      1080
959  ......................................................-................................-................................  AY508462_Elazig_Turkey
959  ......................................................-................................-................................  AY508465_Malatya_Turkey
959  ......................................................-................................-................................  AY508466_Malatya_Turkey
959  ........................G.............................-................................-................................  AY508467_Malatya_Turkey
959  ......................................................-................................-................................  AY508471_Malatya_Turkey
959  ......................................................-................................-................................  AY508472_Malatya_Turkey
959  ......................................................-................................-................................  AY524666_Elazig_Turkey
959  ......................................................-................................-................................  EU073963_Yining_China
959  ......................................................-................................-................................  EU083800_Ningxia_China
959  ......................................................-................................-................................  EU083801_Neimeng_China
959  ......................................................-................................-................................  FJ426369_ITC01_Italy
959  ......................................................-................................-................................  HM538216_China
959  ......................................................-................................-................................  KF429793_Alb3 Alborz_Iran
959  ......................................................-................................-................................  KF429795_Vaccine S15 strain_Iran
959  ......................................................-................................-................................  KF429799_Alb1 Alborz_Iran
959  ......................................................-................................-................................  KF429800_Alb2_Alborz_Iran
959  ......................................................-................................-................................  KF559356_Shanmenxia_China
959  ......................................................-................................-................................  KT367866_Krishna_Andhra Pradesh_India
959  ......................................................-................................-................................  KT367867_Anantapur_Andhra Pradesh_India
959  ......................................................-................................-................................  KT367868_Adilabad_Telangana_India
959  ......................................................-................................-................................  KT367871_Nizamabad_Telangana_India
959  ......................................................-................................-................................  KT367872_Hyderabad_Telangana_India
959  ......................................................-................................-................................  KT367873_Warangal_Telangana_India
959  ......................................................-................................-................................  KT367874_Ranga Reddy_Telangana_India
959  ......................................................-................................-................................  KT367875_Nallagonda_Telangana_India
959  ..A...................................................-................................-..................G.............  KT367876_Chittoor_Andhra Pradesh_India
959  ......................................................-................................-................................  KT367877_Visakhapatnam_Andhra Pradesh_I
959  ......................................................-................................-................................  KT367878_Srikakulam_Andhra Pradesh_Indi
959  ......................................................-................................-...............A................  KT736495_Muzaffarnagar_Uttar Pradesh_In
959  ......................................................-................................-................................  KT736496_Bareilly_Uttar Pradesh_India
959  .............................G........................-................................-................................  KT736497_Bahraich_Uttar Pradesh_India
959  ......................................................-................................-................................  KT736498_Moradabad_Uttar Pradesh_India
958  ......................................................-................................-................................  M64243_Hisar_Haryana_India
959  ...................................................C..-................................-................................  MF287918_Anand_Gujarat 2_India
959  ......................................................-................................-................................  MF287921_Hisar_Haryana 1_India
959  ......................................................-................................-................................  MF287922_Hisar_Haryana 2_India
958  ......................................................-................................-................................  MF287925_Akola_Maharashtra 1_India
959  ......................................................-................................-................................  MF287927_Akola_Maharashtra 3_India
959  ......................................................-................................-................................  MF287929_Bhubaneshwar_Orissa 1_India
960  ..............................................G.......-................................-................................  MF287930_Bhubaneshwar_Orissa 2_India
960  ......................................................-................................-................................  MF287931_Bhubaneshwar_Orissa 3_India
959  ......................................................-................................-................................  MF287932_Bhubaneshwar_Orissa 4_India
959  ......................................................-................................-................................  MF287933_Ludhiana_Punjab 1_India
959  ......................................................-................................-................................  MF287935_Ludhiana_Punjab 3_India
960  ......................................................-................................-................................  MF287936_Ludhiana_Punjab 4_India
959  ......................................................-................................-................................  MF287938_Chennai_Tamil Nadu 2_India
958  ......................................................-................................-................................  MF287941_Udham Singh Nagar_Uttarakhand
959  ......................................................T................................T................................  MF287943_Udham Singh Nagar_Uttarakhand
958  ......................................................-................................-................................  MF287944_Udham Singh Nagar_Uttarakhand
957  ......................................................-................................-................................  MF287945_Bareilly_Uttar Pradesh 1_India
958  ......................................................-................................-................................  MF287946_Bareilly_Uttar Pradesh 2_India
959  ..............................................G.......-................................-................................  MF287947_Bareilly_Uttar Pradesh 3_India
959  ......................................................-................................-................................  MF287951_Bhardhaman_West Bengal 3_India
959  ......................................................-................................-................................  MF287952_Bhardhaman_West Bengal 4_India
```

```
      CCGTTAACGAACGAGACCTTAACCTGCTAAATAGGGTACGGGAATAAGTTTCTACTGTCCCGTTATCGCTTCTTAGAGGGACTTTGCGGTTATAAATCGCAAGGAAGTTTAAGGCAATAA  Majority
               1090      1100      1110      1120      1130      1140      1150      1160      1170      1180      1190      1200
1077  ........................................................................................................................  AY508462_Elazig_Turkey
1077  ........................................................................................................................  AY508465_Malatya_Turkey
1077  ........................................................................................................................  AY508466_Malatya_Turkey
1077  ........................................................................................................................  AY508467_Malatya_Turkey
1077  ........................................................................................................................  AY508471_Malatya_Turkey
1077  ........................................................................................................................  AY508472_Malatya_Turkey
1077  ........................................................................................................................  AY524666_Elazig_Turkey
1077  ........................................................................................................................  EU073963_Yining_China
1077  .....................................................G..................................................................  EU083800_Ningxia_China
1077  ........................................................................................................................  EU083801_Neimeng_China
1077  ........................................................................................................................  FJ426369_ITC01_Italy
1077  ........................................................................................................................  HM538216_China
1077  ........................................................................................................................  KF429793_Alb3 Alborz_Iran
1077  ........................................................................................................................  KF429795_Vaccine S15 strain_Iran
1077  ........................................................................................................................  KF429799_Alb1 Alborz_Iran
1077  ........................................................................................................................  KF429800_Alb2_Alborz_Iran
1077  ........................................................................................................................  KF559356_Shanmenxia_China
1077  ........................................................................................................................  KT367866_Krishna_Andhra Pradesh_India
1077  ...................................................................................................................G....  KT367867_Anantapur_Andhra Pradesh_India
1077  ........................................................................................................................  KT367868_Adilabad_Telangana_India
1077  ........................................................................................................................  KT367871_Nizamabad_Telangana_India
1077  ........................................................................................................................  KT367872_Hyderabad_Telangana_India
1077  ........................................................................................................................  KT367873_Warangal_Telangana_India
1077  ........................................................................................................................  KT367874_Ranga Reddy_Telangana_India
1077  ........................................................................................................................  KT367875_Nallagonda_Telangana_India
1077  .................................................C......................................................................  KT367876_Chittoor_Andhra Pradesh_India
1077  ........................................................................................................................  KT367877_Visakhapatnam_Andhra Pradesh_I
1077  ........................................................................................................................  KT367878_Srikakulam_Andhra Pradesh_Indi
1077  ..........................................................................................................A.............  KT736495_Muzaffarnagar_Uttar Pradesh_In
1077  ........................................................................................................................  KT736496_Bareilly_Uttar Pradesh_India
1077  ........................................................................................................................  KT736497_Bahraich_Uttar Pradesh_India
1077  ........................................................................................................................  KT736498_Moradabad_Uttar Pradesh_India
1076  ........................................................................................................................  M64243_Hisar_Haryana_India
1077  ........................................................................................................................  MF287918_Anand_Gujarat 2_India
1077  ........................................................................................................................  MF287921_Hisar_Haryana 1_India
1077  ........................................................................................................................  MF287922_Hisar_Haryana 2_India
1076  ........................................................................................................................  MF287925_Akola_Maharashtra 1_India
1077  ........................................................................................................................  MF287927_Akola_Maharashtra 3_India
1077  ........................................................................................................................  MF287929_Bhubaneshwar_Orissa 1_India
1078  ........................................................................................................................  MF287930_Bhubaneshwar_Orissa 2_India
1078  ........................................C...............................................................................  MF287931_Bhubaneshwar_Orissa 3_India
1077  ........................................................................................................................  MF287932_Bhubaneshwar_Orissa 4_India
1077  ........................................................................................................................  MF287933_Ludhiana_Punjab 1_India
1077  .........................................................C..............................................................  MF287935_Ludhiana_Punjab 3_India
1078  ........................................................................................................................  MF287936_Ludhiana_Punjab 4_India
1077  ........................................................................................................................  MF287938_Chennai_Tamil Nadu 2_India
1076  ........................................................................................................................  MF287941_Udham Singh Nagar_Uttarakhand
1079  ......................................................................C.................................................  MF287943_Udham Singh Nagar_Uttarakhand
1076  ........................................................................................................................  MF287944_Udham Singh Nagar_Uttarakhand
1075  ........................................................................................................................  MF287945_Bareilly_Uttar Pradesh 1_India
1076  .........................................................C..............................................................  MF287946_Bareilly_Uttar Pradesh 2_India
1077  ........................................................................................................................  MF287947_Bareilly_Uttar Pradesh 3_India
1077  ........................................................................................................................  MF287951_Bhardhaman_West Bengal 3_India
1077  ........................................................................................................................  MF287952_Bhardhaman_West Bengal 4_India
```

```
     CAGGTCTGTGATGCCCTTAGATGTCCTGGGCTGCACGCGCGCTACACTGATGCGTTCATCGAGTTTATCCTTGGCCGAGAGGCCTGGGTAATCTTTAGTACGCATCGTGATGGGGATCGA Majority
              1210      1220      1230      1240      1250      1260      1270      1280      1290      1300      1310      1320
1197 ........................................................................................................................ AY508462_Elazig_Turkey
1197 .....................................................A.................................................................. AY508465_Malatya_Turkey
1197 ........................................................................................................................ AY508466_Malatya_Turkey
1197 ........................................................................................................................ AY508467_Malatya_Turkey
1197 ..................................................C..................................................................... AY508471_Malatya_Turkey
1197 ..................................................................................T..................................... AY508472_Malatya_Turkey
1197 ........................................................................................................................ AY524666_Elazig_Turkey
1197 ........................................................................................................................ EU073963_Yining_China
1197 ........................................................................................................................ EU083800_Ningxia_China
1197 ........................................................................................................................ EU083801_Neimeng_China
1197 ........................................................................................................................ FJ426369_ITC01_Italy
1197 ..................................................C..................................................................... HM538216_China
1197 ........................................................................................................................ KF429793_Alb3 Alborz_Iran
1197 ........................................................................................................................ KF429795_Vaccine S15 strain_Iran
1197 ........................................................................................................................ KF429799_Alb1 Alborz_Iran
1197 ........................................................................................................................ KF429800_Alb2_Alborz_Iran
1197 ........................................................................................................................ KF559356_Shanmenxia_China
1197 ........................................................................................................................ KT367866_Krishna_Andhra Pradesh_India
1197 ........................................................................................................................ KT367867_Anantapur_Andhra Pradesh_India
1197 ........................................................................................................................ KT367868_Adilabad_Telangana_India
1197 ........................................................................................................................ KT367871_Nizamabad_Telangana_India
1197 ........................................................................................................................ KT367872_Hyderabad_Telangana_India
1197 ........................................................................................................................ KT367873_Warangal_Telangana_India
1197 ........................................................................................................................ KT367874_Ranga Reddy_Telangana_India
1197 ........................................................................................................................ KT367875_Nallagonda_Telangana_India
1197 ........................................................................................................................ KT367876_Chittoor_Andhra Pradesh_India
1197 ........................................................................................................................ KT367877_Visakhapatnam_Andhra Pradesh_I
1197 ..................................................................................................C..................... KT367878_Srikakulam_Andhra Pradesh_Indi
1197 ........................................................................................................................ KT736495_Muzaffarnagar_Uttar Pradesh_In
1197 ..................................................................C..................................................... KT736496_Bareilly_Uttar Pradesh_India
1197 ........................................................................................................................ KT736497_Bahraich_Uttar Pradesh_India
1197 ........................................................................................................................ KT736498_Moradabad_Uttar Pradesh_India
1196 ........................................................................................................................ M64243_Hisar_Haryana_India
1197 ........................................................................................................................ MF287918_Anand_Gujarat 2_India
1197 ........................................................................................................................ MF287921_Hisar_Haryana 1_India
1197 ........................................................................................................................ MF287922_Hisar_Haryana 2_India
1196 ........................................................................................................................ MF287925_Akola_Maharashtra 1_India
1197 ........................................................................................................................ MF287927_Akola_Maharashtra 3_India
1197 ........................................................................................................................ MF287929_Bhubaneshwar_Orissa 1_India
1198 ........................................................................................................................ MF287930_Bhubaneshwar_Orissa 2_India
1198 ........................................................................................................................ MF287931_Bhubaneshwar_Orissa 3_India
1197 ........................................................................................................................ MF287932_Bhubaneshwar_Orissa 4_India
1197 ........................................................................................................................ MF287933_Ludhiana_Punjab 1_India
1197 ........................................................................................................................ MF287935_Ludhiana_Punjab 3_India
1198 ........................................................................................................................ MF287936_Ludhiana_Punjab 4_India
1197 ........................................................................................................................ MF287938_Chennai_Tamil Nadu 2_India
1196 ........................................................................................................................ MF287941_Udham Singh Nagar_Uttarakhand
1199 ........................................................................................................................ MF287943_Udham Singh Nagar_Uttarakhand
1196 ........................................................................................................................ MF287944_Udham Singh Nagar_Uttarakhand
1195 ........................................................................................................................ MF287945_Bareilly_Uttar Pradesh 1_India
1196 ........................................................................................................................ MF287946_Bareilly_Uttar Pradesh 2_India
1197 ........................................................................................................................ MF287947_Bareilly_Uttar Pradesh 3_India
1197 ........................................................................................................................ MF287951_Bhardhaman_West Bengal 3_India
1197 ........................................................................................................................ MF287952_Bhardhaman_West Bengal 4_India

     TTATTGCAATTGTTAATCGTGAACGAGGAATGCCTAGTATGCGCAAGTCATCAGCTTGTGCAGATTACGTCCCTGCCCTTTGTACACACCGCCCGTCGCTCCTACCGATCGAGTGATCCG Majority
              1330      1340      1350      1360      1370      1380      1390      1400      1410      1420      1430      1440
1317 ........................................................................................................................ AY508462_Elazig_Turkey
1317 ........................................................................................................................ AY508465_Malatya_Turkey
1317 ........................................................................................................................ AY508466_Malatya_Turkey
1317 .................................................G........................................A............................. AY508467_Malatya_Turkey
1317 ........................................................................................................................ AY508471_Malatya_Turkey
1317 ........................................................................................................................ AY508472_Malatya_Turkey
1317 ........................................................................................................................ AY524666_Elazig_Turkey
1317 ........................................................................................................................ EU073963_Yining_China
1317 .....................................................................C.................................................. EU083800_Ningxia_China
1317 ........................................................................................................................ EU083801_Neimeng_China
1317 ........................................................................................................................ FJ426369_ITC01_Italy
1317 ........................................................................................................................ HM538216_China
1317 ........................................................................................................................ KF429793_Alb3 Alborz_Iran
1317 ........................................................................................................................ KF429795_Vaccine S15 strain_Iran
1317 ........................................................................................................................ KF429799_Alb1 Alborz_Iran
1317 ........................................................................................................................ KF429800_Alb2_Alborz_Iran
1317 ........................................................................................................................ KF559356_Shanmenxia_China
1317 ........................................................................................................................ KT367866_Krishna_Andhra Pradesh_India
1317 ........................................................................................................................ KT367867_Anantapur_Andhra Pradesh_India
1317 ........................................................................................................................ KT367868_Adilabad_Telangana_India
1317 ........................................................................................................................ KT367871_Nizamabad_Telangana_India
1317 ........................................................................................................................ KT367872_Hyderabad_Telangana_India
1317 ........................................................................................................................ KT367873_Warangal_Telangana_India
1317 ........................................................................................................................ KT367874_Ranga Reddy_Telangana_India
1317 ........................................................................................................................ KT367875_Nallagonda_Telangana_India
1317 ........................................................................................................................ KT367876_Chittoor_Andhra Pradesh_India
1317 ........................................................................................................................ KT367877_Visakhapatnam_Andhra Pradesh_I
1317 ........................................................................................................................ KT367878_Srikakulam_Andhra Pradesh_Indi
1317 ........................................................................................................................ KT736495_Muzaffarnagar_Uttar Pradesh_In
1317 ........................................................................................................................ KT736496_Bareilly_Uttar Pradesh_India
1317 ........................................................................................................................ KT736497_Bahraich_Uttar Pradesh_India
1317 ........................................................................................................................ KT736498_Moradabad_Uttar Pradesh_India
1316 ........................................................................................................................ M64243_Hisar_Haryana_India
1317 ........................................................................................................................ MF287918_Anand_Gujarat 2_India
1317 ........................................................................................................................ MF287921_Hisar_Haryana 1_India
1317 ........................................................................................................................ MF287922_Hisar_Haryana 2_India
1316 ........................................................................................................................ MF287925_Akola_Maharashtra 1_India
1317 ........................................................................................................................ MF287927_Akola_Maharashtra 3_India
1317 ........................................................................................................................ MF287929_Bhubaneshwar_Orissa 1_India
1318 ........................................................................................................................ MF287930_Bhubaneshwar_Orissa 2_India
1318 ...............................................................................C........................................ MF287931_Bhubaneshwar_Orissa 3_India
1317 ........................................................................................................................ MF287932_Bhubaneshwar_Orissa 4_India
1317 ........................................................................................................................ MF287933_Ludhiana_Punjab 1_India
1317 ........................................................................................................................ MF287935_Ludhiana_Punjab 3_India
1318 ........................................................................................................................ MF287936_Ludhiana_Punjab 4_India
1317 ........................................................................................................................ MF287938_Chennai_Tamil Nadu 2_India
1316 ........................................................................................................................ MF287941_Udham Singh Nagar_Uttarakhand
1319 ........................................................................................................................ MF287943_Udham Singh Nagar_Uttarakhand
1316 ........................................................................................................................ MF287944_Udham Singh Nagar_Uttarakhand
1315 ........................................................................................................................ MF287945_Bareilly_Uttar Pradesh 1_India
1316 ........................................................................................................................ MF287946_Bareilly_Uttar Pradesh 2_India
1317 ........................................................................................................................ MF287947_Bareilly_Uttar Pradesh 3_India
1317 ........................................................................................................................ MF287951_Bhardhaman_West Bengal 3_India
1317 ........................................................................................................................ MF287952_Bhardhaman_West Bengal 4_India
```

	GTGAATTATTCGGACCGTGATGTTCCCGTCAGGGAACGTCTAGGGAAGTTTTGTGAACCTTATCACTTAAAGGAAGGAGAAGTCGTAACAAGG	Majority
	1450 1460 1470 1480 1490 1500 1510 1520 1530	
1437	....	AY508462_Elazig_Turkey
1437	....C..........	AY508465_Malatya_Turkey
1437	....	AY508466_Malatya_Turkey
1437	....	AY508467_Malatya_Turkey
1437	....	AY508471_Malatya_Turkey
1437	....	AY508472_Malatya_Turkey
1437	....	AY524666_Elazig_Turkey
1437	....	EU073963_Yining_China
1437	....	EU083800_Ningxia_China
1437	....	EU083801_Neimeng_China
1437	....	FJ426369_ITC01_Italy
1437	....	HM538216_China
1437	....	KF429793_Alb3 Alborz_Iran
1437	....	KF429795_Vaccine S15 strain_Iran
1437	....	KF429799_Alb1 Alborz_Iran
1437	....	KF429800_Alb2_Alborz_Iran
1437	....	KF559356_Shanmenxia_China
1437	....	KT367866_Krishna_Andhra Pradesh_India
1437	....	KT367867_Anantapur_Andhra Pradesh_India
1437	....	KT367868_Adilabad_Telangana_India
1437	....	KT367871_Nizamabad_Telangana_India
1437	....	KT367872_Hyderabad_Telangana_India
1437	....	KT367873_Warangal_Telangana_India
1437	....	KT367874_Ranga Reddy_Telangana_India
1437	....	KT367875_Nallagonda_Telangana_India
1437	..................................G..	KT367876_Chittoor_Andhra Pradesh_India
1437	..................................G..	KT367877_Visakhapatnam_Andhra Pradesh_I
1437	....	KT367878_Srikakulam_Andhra Pradesh_Indi
1437	....G.....................................	KT736495_Muzaffarnagar_Uttar Pradesh_In
1437	...................T..	KT736496_Bareilly_Uttar Pradesh_India
1437	........C..	KT736497_Bahraich_Uttar Pradesh_India
1437	....	KT736498_Moradabad_Uttar Pradesh_India
1436	....	M64243_Hisar_Haryana_India
1437	....	MF287918_Anand_Gujarat 2_India
1437	....	MF287921_Hisar_Haryana 1_India
1437	...................G.........................G...	MF287922_Hisar_Haryana 2_India
1436	........................T..	MF287925_Akola_Maharashtra 1_India
1437	...................G.........................G...	MF287927_Akola_Maharashtra 3_India
1437	....	MF287929_Bhubaneshwar_Orissa 1_India
1438	....	MF287930_Bhubaneshwar_Orissa 2_India
1438	....	MF287931_Bhubaneshwar_Orissa 3_India
1437	....	MF287932_Bhubaneshwar_Orissa 4_India
1437	....	MF287933_Ludhiana_Punjab 1_India
1437	....	MF287935_Ludhiana_Punjab 3_India
1438	....	MF287936_Ludhiana_Punjab 4_India
1437	....	MF287938_Chennai_Tamil Nadu 2_India
1436	....	MF287941_Udham Singh Nagar_Uttarakhand
1439	....	MF287943_Udham Singh Nagar_Uttarakhand
1436	....	MF287944_Udham Singh Nagar_Uttarakhand
1435	....	MF287945_Bareilly_Uttar Pradesh 1_India
1436	....	MF287946_Bareilly_Uttar Pradesh 2_India
1437	.....C.......G.....................................A...	MF287947_Bareilly_Uttar Pradesh 3_India
1437	....	MF287951_Bhardhaman_West Bengal 3_India
1437	....	MF287952_Bhardhaman_West Bengal 4_India

Decoration 'Decoration #1': Hide (as '.') residues that match the Consensus exactly.

Decoration 'Decoration #2': Shade (with solid deep red) residues that differ from the Consensus.
