# Supplementary material for: An insight into misidentification of the small-subunit ribosomal RNA (18S rRNA) gene sequences of Theileria spp. as Theileria annulata
Source: BMC Vet Res. 2022 Dec 28;18:454. doi: 10.1186/s12917-022-03540-w (PMC9795727; doi:10.1186/s12917-022-03540-w)
Supplement: Supplementary file 4 — Additional file 4. Supplementary File 2: Multiple sequence alignment of the nearly complete 18S rRNA gene sequences of T. orientalis group used in the sequence and phylogenetic analyses in the present study. [file 12917_2022_3540_MOESM4_ESM.pdf]

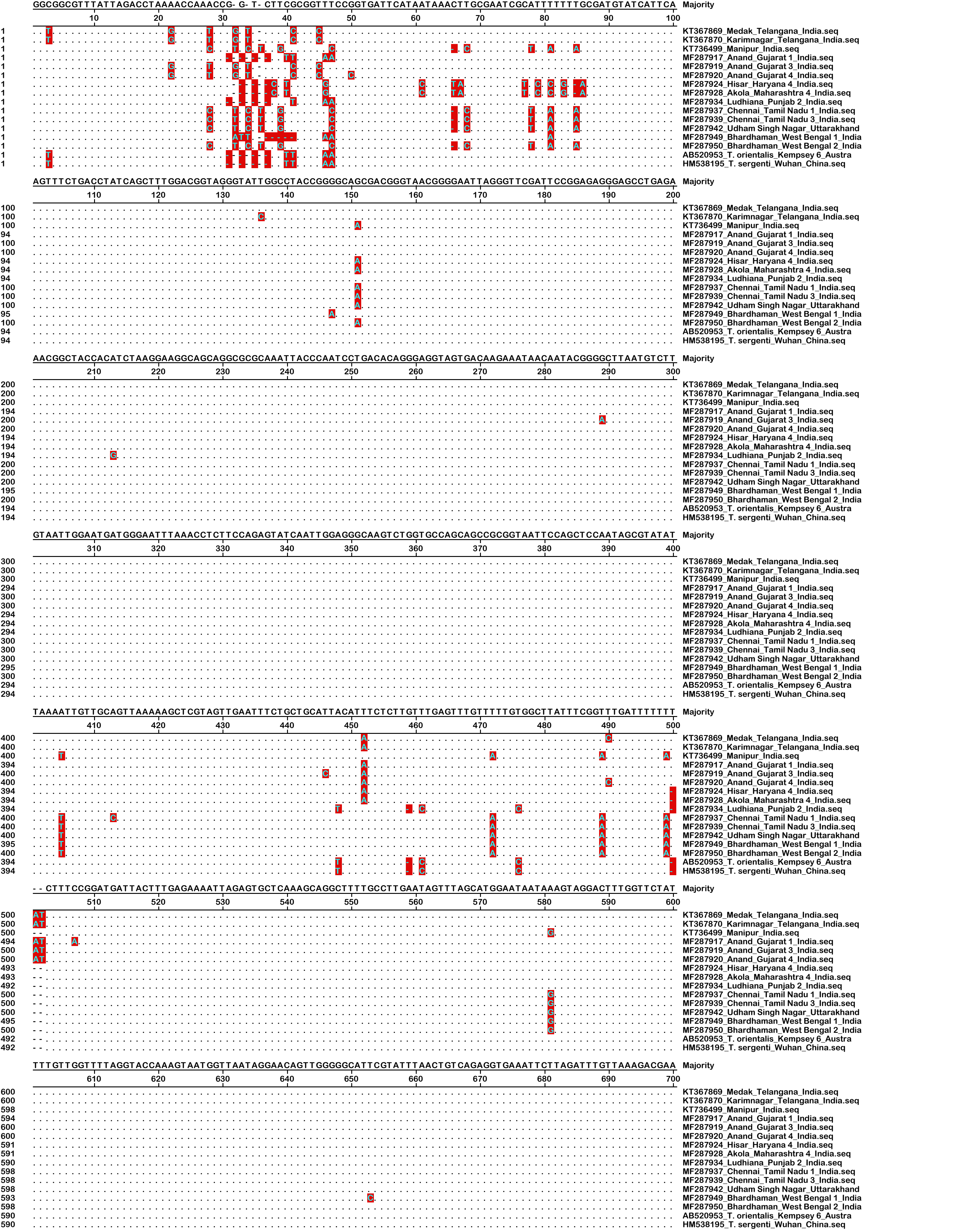

GGCGGCGTTTATTAGACCTAAAACCAAACCG- G- T- CTTCGCGGTTTCCGGTGATTCATAATAAACTTGCGAATCGCATTTTTTTGCGATGTATCATTCA Majority
AGTTTCTGACCTATCAGCTTTGGACGGTAGGGTATTGGCCTACCGGGGCAGCGACGGGTAACGGGGAATTAGGGTTCGATTCCGGAGAGGGAGCCTGAGA Majority
AACGGCTACCACATCTAAGGAAGGCAGCAGGCGCGCAAATTACCCAATCCTGACACAGGGAGGTAGTGACAAGAAATAACAATACGGGGCTTAATGTCTT Majority
GTAATTGGAATGATGGGAATTTAAACCTCTTCCAGAGTATCAATTGGAGGGCAAGTCTGGTGCCAGCAGCCGCGGTAATTCCAGCTCCAATAGCGTATAT Majority
TAAAATTGTTGCAGTTAAAAAGCTCGTAGTTGAATTTCTGCTGCATTACATTTCTCTTGTTTGAGTTTGTTTTTGTGGCTTATTTCGGTTTGATTTTTTT Majority
- - CTTTCCGGATGATTACTTTGAGAAAATTAGAGTGCTCAAAGCAGGCTTTTGCCTTGAATAGTTTAGCATGGAATAATAAAGTAGGACTTGGTTCTAT Majority
TTTGTTGGTTTTAGGTACCAAAGTAATGGTTAATAGGAACAGTTGGGGGCATTCGTATTTAACTGTCAGAGGTGAAATTCTTAGATTTGTTAAAGACGAA Majority
KT367869_Medak_Telangana_India.seq
KT367870_Karimnagar_Telangana_India.seq
KT736499_Manipur_India.seq
MF287917_Anand_Gujarat 1_India.seq
MF287919_Anand_Gujarat 3_India.seq
MF287920_Anand_Gujarat 4_India.seq
MF287924_Hisar_Haryana 4_India.seq
MF287928_Akola_Maharashtra 4_India.seq
MF287934_Ludhiana_Punjab 2_India.seq
MF287937_Chennai_Tamil Nadu 1_India.seq
MF287939_Chennai_Tamil Nadu 3_India.seq
MF287942_Udham Singh Nagar_Uttarakhand
MF287949_Bhardhaman_West Bengal 1_India
MF287950_Bhardhaman_West Bengal 2_India
AB520953_T. orientalis_Kempsey 6_Austra
HM538195_T. sergenti_Wuhan_China.seq

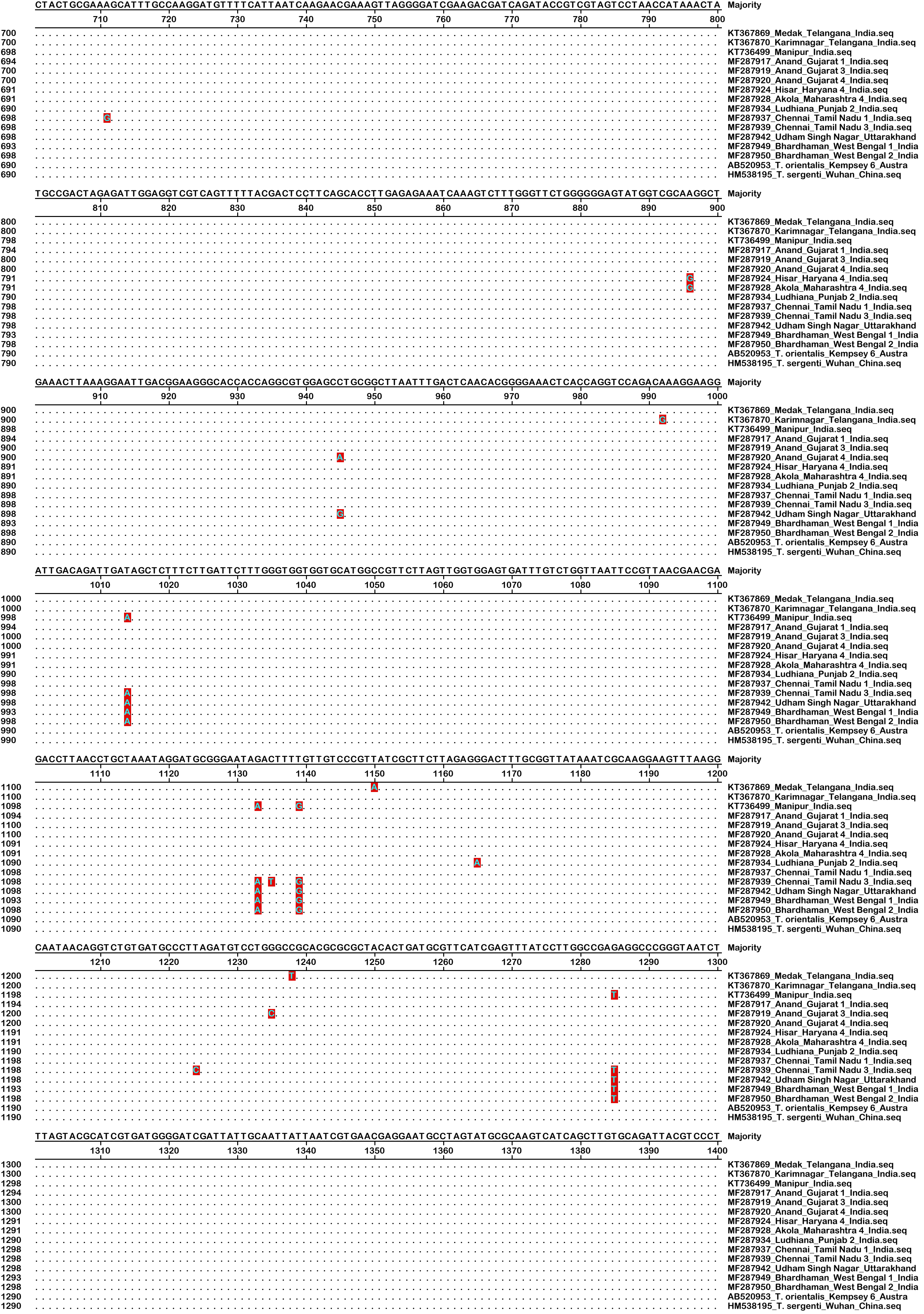

CTACTGCGAAAGCATTTGCCAAGGATGTTTTCATTAATCAAGAACGAAAGTTAGGGGATCGAAGACGATCAGATACCGTCGTAGTCCTAACCATAAACTA Majority
TGCCGACTAGAGATTGGAGGTCGTCAGTTTTTACGACTCCTTCAGCACCTTGAGAGAAATCAAAGTCTTTGGGTTCTGGGGGGAGTATGGTCGCAAGGCT Majority
GAAACTTAAAGGAATTGACGGAAGGGCACCACCAGGCGTGGAGCCTGCGGCTTAATTTGACTCAACACGGGGAAACTCACCAGGTCCAGACAAAGGAAGG Majority
ATTGACAGATTGATAGCTCTTTCTTGATTCTTTGGGTGGTGGTGCATGGCCGTTCTTAGTTGGTGGAGTGATTTGTCTGGTTAATTCCGTTAACGAACGA Majority
GACCTTAACCTGCTAAATAGGATGCGGGAATAGACTTTTGTTGTCCCGTTATCGCTTCTTAGAGGGACTTTGCGGTTATAAATCGCAAGGAAGTTTAAGG Majority
CAATAACAGGTCTGTGATGCCCTTAGATGTCCTGGGCCGCACGCGCGCTACACTGATGCGTTCATCGAGTTTATCCTTGGCCGAGAGGCCCGGGTAATCT Majority
TTAGTACGCATCGTGATGGGGATCGATTATTGCAATTATTAATCGTGAACGAGGAATGCCTAGTATGCGCAAGTCATCAGCTTGTGCAGATTACGTCCCT Majority
KT367869_Medak_Telangana_India.seq
KT367870_Karimnagar_Telangana_India.seq
KT736499_Manipur_India.seq
MF287917_Anand_Gujarat 1_India.seq
MF287919_Anand_Gujarat 3_India.seq
MF287920_Anand_Gujarat 4_India.seq
MF287924_Hisar_Haryana 4_India.seq
MF287928_Akola_Maharashtra 4_India.seq
MF287934_Ludhiana_Punjab 2_India.seq
MF287937_Chennai_Tamil Nadu 1_India.seq
MF287939_Chennai_Tamil Nadu 3_India.seq
MF287942_Udham Singh Nagar_Uttarakhand
MF287949_Bhardhaman_West Bengal 1_India
MF287950_Bhardhaman_West Bengal 2_India
AB520953_T. orientalis_Kempsey 6_Austra
HM538195_T. sergenti_Wuhan_China.seq

```
     GCCCTTTGTACACACCGCCCGTCGCTCCTACCGATCGAGTGATCCGGTGAATTATTCGGACCGTGATGTTCCCGTTAGGGAACGTCTAGGGAAGTTTTGT  Majority
              1410      1420      1430      1440      1450      1460      1470      1480      1490      1500
1400 ...........................................................................C........................  KT367869_Medak_Telangana_India.seq
1400 ....................................................................................................  KT367870_Karimnagar_Telangana_India.seq
1398 ...........................................................................C........................  KT736499_Manipur_India.seq
1394 ....................................................................................................  MF287917_Anand_Gujarat 1_India.seq
1400 ..........................................................................C.........................  MF287919_Anand_Gujarat 3_India.seq
1400 ....................................................................................................  MF287920_Anand_Gujarat 4_India.seq
1391 ....................................................................................................  MF287924_Hisar_Haryana 4_India.seq
1391 ....................................................................................................  MF287928_Akola_Maharashtra 4_India.seq
1390 ....................................................................................................  MF287934_Ludhiana_Punjab 2_India.seq
1398 .................................................................................G..................  MF287937_Chennai_Tamil Nadu 1_India.seq
1398 ...........................................................................C........................  MF287939_Chennai_Tamil Nadu 3_India.seq
1398 ...........................................................................C........................  MF287942_Udham Singh Nagar_Uttarakhand
1393 ...........................................................................C........................  MF287949_Bhardhaman_West Bengal 1_India
1398 ...........................................................................C........................  MF287950_Bhardhaman_West Bengal 2_India
1390 ....................................................................................................  AB520953_T. orientalis_Kempsey 6_Austra
1390 ....................................................................................................  HM538195_T. sergenti_Wuhan_China.seq

     GAACCTTATCACTTAAAGGAAGGAGAAGTCGTAACAAGG  Majority
              1510      1520      1530
1500 .......................................  KT367869_Medak_Telangana_India.seq
1500 .......................................  KT367870_Karimnagar_Telangana_India.seq
1498 .......................................  KT736499_Manipur_India.seq
1494 .......................................  MF287917_Anand_Gujarat 1_India.seq
1500 .......................................  MF287919_Anand_Gujarat 3_India.seq
1500 .......................................  MF287920_Anand_Gujarat 4_India.seq
1491 .......................................  MF287924_Hisar_Haryana 4_India.seq
1491 .......................................  MF287928_Akola_Maharashtra 4_India.seq
1490 .......................................  MF287934_Ludhiana_Punjab 2_India.seq
1498 .......................................  MF287937_Chennai_Tamil Nadu 1_India.seq
1498 .......................................  MF287939_Chennai_Tamil Nadu 3_India.seq
1498 ..G....................................  MF287942_Udham Singh Nagar_Uttarakhand
1493 .......................................  MF287949_Bhardhaman_West Bengal 1_India
1498 ..G....................................  MF287950_Bhardhaman_West Bengal 2_India
1490 .......................................  AB520953_T. orientalis_Kempsey 6_Austra
1490 .......................................  HM538195_T. sergenti_Wuhan_China.seq
```

Decoration 'Decoration #1': Hide (as '.') residues that match the Consensus exactly.

Decoration 'Decoration #2': Shade (with solid deep red) residues that differ from the Consensus.
